# Correction: Factors influencing appropriate use of interventions for management of women experiencing preterm birth: A mixed-methods systematic review and narrative synthesis

**DOI:** 10.1371/journal.pmed.1004105

**Published:** 2022-09-22

**Authors:** Rana Islamiah Zahroh, Alya Hazfiarini, Katherine E. Eddy, Joshua P. Vogel, Ӧzge Tunçalp, Nicole Minckas, Fernando Althabe, Olufemi T. Oladapo, Meghan A. Bohren

The copyright information is incorrect. The correct copyright details for this article are as follows:

© 2022 World Health Organization. Licensee Public Library of Science. This is an open access article distributed under the Creative Commons Attribution IGO License, which permits unrestricted use, distribution, and reproduction in any medium, provided the original work is properly cited. http://creativecommons.org/licenses/by/3.0/igo/. In any use of this article, there should be no suggestion that WHO endorses any specific organisation, products or services. The use of the WHO logo is not permitted. This notice should be preserved along with the article’s original URL.
