# Supplementary material for: Factors influencing appropriate use of interventions for management of women experiencing preterm birth: A mixed-methods systematic review and narrative synthesis
Source: PLoS Med. 2022 Aug 23;19(8):e1004074. doi: 10.1371/journal.pmed.1004074 (PMC9398034; doi:10.1371/journal.pmed.1004074)
Supplement: S2 Appendix — (PDF) [file pmed.1004074.s002.pdf]

S2 Appendix. ENTREQ Reporting Checklist

No	Item	Guide and description	Location where item reported
**1**	Aim	State the research question the synthesis addresses	Introduction: Paragraph 3
**2**	Synthesis methodology	Identify the synthesis methodology or theoretical framework which underpins the synthesis, and describe the rationale for choice of methodology (e.g., meta-ethnography, thematic synthesis, critical interpretive synthesis, grounded theory synthesis, realist synthesis, meta-aggregation, meta-study, framework synthesis).	Methods: Data management, analysis, and synthesis – paragraph 1
**3**	Approach to searching	Indicate whether the search was pre-planned (comprehensive search strategies to seek all available studies) or iterative (to seek all available concepts until they theoretical saturation is achieved).	Methods: Search methods for identification of studies – paragraph 1
**4**	Inclusion criteria	Specify the inclusion/exclusion criteria (e.g., in terms of population, language, year limits, type of publication, study type)	Methods: Type of studies – paragraph 1 and Topic of interest – paragraph 1
**5**	Data sources	Describe the information sources used (e.g., electronic databases (MEDLINE, EMBASE, CINAHL, psycINFO, Econlit), grey literature databases (digital thesis, policy reports), relevant organisational websites, experts, information specialists, generic web searches (Google Scholar) hand searching, reference lists) and when the searches conducted; provide the rationale for using the data sources	Methods: Search methods for identification of studies – paragraph 1
**6**	Electronic Search strategy	Describe the literature search (e.g., provide electronic search strategies with population terms, clinical or health topic terms, experiential or social phenomena related terms, filters for qualitative research, and search limits)	Methods: Search methods for identification of studies – paragraph 1
**7**	Study screening methods	Describe the process of study screening and sifting (e.g., title, abstract and full text review, number of independent reviewers who screened studies).	Methods: Selection of studies – paragraph 1
**8**	Study characteristics	Present the characteristics of the included studies (e.g., year of publication, country, population, number of participants, data collection, methodology, analysis, research questions).	Results paragraph 2-5 and Appendix S7
**9**	Study selection results	Identify the number of studies screened and provide reasons for study exclusion (egg, for comprehensive searching, provide numbers of studies screened and reasons for exclusion indicated in a figure/flowchart; for iterative searching describe reasons for study exclusion and inclusion based on modifications to the research question and/or contribution to theory development).	Results paragraph 1 and Figure 2. PRISMA flowchart
**10**	Rationale for appraisal	Describe the rationale and approach used to appraise the included studies or selected findings (e.g., assessment of conduct (validity and robustness), assessment of reporting (transparency), assessment of content and utility of the findings).	Methods: Data extraction and assessing methodological limitations – paragraph 2

No	Item	Guide and description	Location where item reported
**11**	Appraisal items	State the tools, frameworks and criteria used to appraise the studies or selected findings (e.g., Existing tools: CASP, QARI, COREQ, Mays and Pope [25]; reviewer developed tools; describe the domains assessed: research team, study design, data analysis and interpretations, reporting).	Methods: Data extraction and assessing methodological limitations – paragraph 2
**12**	Appraisal process	Indicate whether the appraisal was conducted independently by more than one reviewer and if consensus was required	Methods: Data extraction and assessing methodological limitations – paragraph 2
**13**	Appraisal results	Present results of the quality assessment and indicate which articles, if any, were weighted/excluded based on the assessment and give the rationale.	Results paragraph 6 and Appendix S4
**14**	Data extraction	Indicate which sections of the primary studies were analysed and how were the data extracted from the primary studies? (e.g., all text under the headings “results /conclusions” were extracted electronically and entered into a computer software).	Methods: Data extraction and assessing methodological limitations – paragraph 1
**15**	Software	State the computer software used if any	Methods: Data management, analysis, and synthesis – paragraph 1
**16**	Number of reviewers	Identify who was involved in coding and analysis.	Methods: Data management, analysis, and synthesis – paragraph 1
**17**	Coding	Describe the process for coding of data (e.g., line by line coding to search for concepts)	Methods: Data management, analysis, and synthesis – paragraph 1
**18**	Study comparison	Describe how were comparisons made within and across studies (e.g., subsequent studies were coded into pre-existing concepts, and new concepts were created when deemed necessary)	Methods: Data management, analysis, and synthesis – paragraph 1-2
**19**	Derivation of themes	Explain whether the process of deriving the themes or constructs was inductive or deductive.	Methods: Data management, analysis, and synthesis – paragraph 1 and Appendix S9
**20**	Quotations	Provide quotations from the primary studies to illustrate themes/constructs and identify whether the quotations were participant quotations of the author’s interpretation.	Quotations not provided as this is qualitative evidence synthesis
**21**	Synthesis output	Present rich, compelling, and useful results that go beyond a summary of the primary studies (e.g., new interpretation, models of evidence, conceptual models, analytical framework, development of a new theory or construct).	All paragraph under “Results of qualitative and quantitative synthesis” sub-heading on the results section
