# Supplementary material for: Factors influencing appropriate use of interventions for management of women experiencing preterm birth: A mixed-methods systematic review and narrative synthesis
Source: PLoS Med. 2022 Aug 23;19(8):e1004074. doi: 10.1371/journal.pmed.1004074 (PMC9398034; doi:10.1371/journal.pmed.1004074)
Supplement: S3 Appendix — (PDF) [file pmed.1004074.s003.pdf]

S3 Appendix. Search strategies

Database: Medline (Ovid)

#	Searches
**1**	(pregnan* or antenatal or ante-natal or prenatal).mp.
**2**	(preterm birth* or pre-term birth* or premature birth* or prematur*).mp.
**3**	(adrenal cortex hormone* or corticosteroid* or corticoid* or glucocorticoid* or steroid* or betamethasone or dexamethasone or hydrocortisone).mp.
**4**	1 and 2 and 3
**5**	(tocolytic* or tocolysis*or betamimetic* or beta agonist or terbutaline or ritodrine or isoxsuprine or fenoterol or hexoprenaline or salbutamol or albuterol or calcium channel blocker* or nifedipine or nicardipine or verapamil or cyclo-oxygenase inhibitor* or cox inhibitor* or cox-2 inhibitor* or indomethacin or nimesulide or sulindac or ketorolac or rofecoxib or celecoxib or naproxen or magnesium sulphate or magnesium sulfate or mgso4 or magnesium gluconate* or epsom salt* or oxytocin receptor antagonist* or atosiban or barusiban or nitric oxide donor* or nitroglycerin or isosorbide dinitrate or progestational agent* or progesterone or relaxin).mp.
**6**	1 and 2 and 5
**7**	(magnesium sulphate or magnesium sulfate or mgso4 or magnesium gluconate* or epsom salt*).mp.
**8**	(fetal neuroprotect* or foetal neuroprotecti*).mp.
**9**	1 and 2 and 7 and 8
**10**	(antibiotic* or penicillin* or anti-bacterial agent* or beta lactam or macrolide or ampicillin or amoxicillin clavulanic acid or co-amoxiclav or benzylpenicillin or metzlocillin or erythromycin or penicillin or ampicillin sulbactam or piperacillin or clindamycin ormetronidazole or azithromycin or cefazolin or clarithromycin or gentamycin or sulbactam).mp.
**11**	(preterm prelabor rupture of membranes or preterm prelabour rupture of membranes or pre-term prelabor rupture of membranes or pre-term prelabour rupture of membranes or pprom or preterm rupture of membranes or pre-term rupture of membranes).mp.
**12**	1 and 2 and 10 and 11
**13**	4 or 6 or 9 or 12
**14**	limit 13 to humans

Database: EMBASE (Ovid)

#	Searches
**1**	(pregnan* or antenatal or ante-natal or prenatal).mp.
**2**	(preterm birth* or pre-term birth* or premature birth* or prematur*).mp.
**3**	(adrenal cortex hormone* or corticosteroid* or corticoid* or glucocorticoid* or steroid* or betamethasone or dexamethasone or hydrocortisone).mp.
**4**	1 and 2 and 3
**5**	(tocolytic* or tocolysis*or betamimetic* or beta agonist or terbutaline or ritodrine or isoxsuprine or fenoterol or hexoprenaline or salbutamol or albuterol or calcium channel blocker* or nifedipine or nicardipine or verapamil or cyclo-oxygenase inhibitor* or cox inhibitor* or cox-2 inhibitor* or indomethacin or nimesulide or sulindac or ketorolac or rofecoxib or celecoxib or naproxen or magnesium sulphate or magnesium sulfate or mgso4 or magnesium gluconate* or epsom salt* or oxytocin receptor antagonist* or atosiban or barusiban or nitric oxide donor* or nitroglycerin or isosorbide dinitrate or progestational agent* or progesterone or relaxin).mp.
**6**	1 and 2 and 5
**7**	(magnesium sulphate or magnesium sulfate or mgso4 or magnesium gluconate* or epsom salt*).mp.
**8**	(fetal neuroprotect* or foetal neuroprotecti*).mp.
**9**	1 and 2 and 7 and 8
**10**	(antibiotic* or penicillin* or anti-bacterial agent* or beta lactam or macrolide or ampicillin or amoxicillin clavulanic acid or co-amoxiclav or benzylpenicillin or metzlocillin or erythromycin or penicillin or ampicillin sulbactam or piperacillin or clindamycin ormetronidazole or azithromycin or cefazolin or clarithromycin or gentamycin or sulbactam).mp.
**11**	(preterm prelabor rupture of membranes or preterm prelabour rupture of membranes or pre-term prelabor rupture of membranes or pre-term prelabour rupture of membranes or pprom or preterm rupture of membranes or pre-term rupture of membranes).mp.
**12**	1 and 2 and 10 and 11
**13**	4 or 6 or 9 or 12
**14**	limit 13 to (human and exclude medline journals and (article or article in press))

Database: CINAHL COMPLETE

#	Searches
**1**	(pregnan* or antenatal or ante-natal or prenatal)
**2**	(“preterm birth*” or “pre-term birth*” or “premature birth*” or prematur*)
**3**	(“adrenal cortex hormone*” or corticosteroid* or corticoid* or glucocorticoid* or steroid* or betamethasone or dexamethasone or hydrocortisone)
**4**	S1 and S2 and S3
**5**	(tocolytic* or tocolysis*or betamimetic* or “beta agonist” or terbutaline or ritodrine or isoxsuprine or fenoterol or hexoprenaline or salbutamol or albuterol or “calcium channel blocker*” or nifedipine or nicardipine or verapamil or “cyclo-oxygenase inhibitor*” or “cox inhibitor*” or “cox-2 inhibitor*” or indomethacin or nimesulide or sulindac or ketorolac or rofecoxib or celecoxib or naproxen or “magnesium sulphate” or “magnesium sulfate” or mgso4 or “magnesium gluconate*” or “epsom salt*” or “oxytocin receptor antagonist*” or atosiban or barusiban or “nitric oxide donor*” or nitroglycerin or “isosorbide dinitrate” or “progestational agent*” or “progesterone or relaxin”)
**6**	S1 and S2 and S5
**7**	(“magnesium sulphate” or “magnesium sulfate” or mgso4 or “magnesium gluconate*” or “epsom salt*”)
**8**	(“fetal neuroprotect*” or “foetal neuroprotecti*”)
**9**	S1 and S2 and S7 and S8
**10**	(antibiotic* or penicillin* or “anti-bacterial agent*” or “beta lactam” or macrolide or ampicillin or “amoxicillin clavulanic acid” or co-amoxiclav or benzylpenicillin or metzlocillin or erythromycin or penicillin or “ampicillin sulbactam” or piperacillin or “clindamycin ormetronidazole” or azithromycin or cefazolin or clarithromycin or gentamycin or sulbactam)
**11**	(“preterm prelabor rupture of membranes” or “preterm prelabour rupture of membranes” or “pre-term prelabor rupture of membranes” or “pre-term prelabour rupture of membranes” or pprom or “preterm rupture of membranes” or “pre-term rupture of membranes”)
**12**	S1 and S2 and S10 and S11
**13**	S4 or S6 or S9 or S12

Database: Global health

We put the following script on the search box and set limits to human by using the 'Refine by' filters on the right-hand column. Refine by: Organism Descriptors > select 'Show List' > Select 'man' > 'Refine Results'.

Searches
((("preterm prelabor rupture of membranes" or "preterm prelabour rupture of membranes" or "pre-term prelabor ruptur of membranes" or "pre-term prelabour rupture of membranes" or pprom or "preterm rupture of membranes" or "pre-term rupture of membranes")) AND ((antibiotic* or penicillin* or "anti-bacterial agent*" or "beta lactam" or macrolide or ampicillin or "amoxicillin clavulanic acid" or co-amoxiclav or benzylpenicillin or metzlocillin or erythromycin or penicillin or "ampicillin sulbactam" or piperacillin or "clindamycin ormetronidazole" or azithromycin or cefazolin or clarithromycin or gentamycin or sulbactam)) AND ("preterm birth*" or "pre-term birth*" or "premature birth*" or prematur*) AND ((pregnan* or antenatal or "ante-natal" or prenatal))) OR ((("fetal neuroprotect*" or "foetal neuroprotecti*")) AND ("magnesium sulphate" or "magnesium sulfate" or mgso4 or "magnesium gluconate*" or "epsom salt*") AND ("preterm birth*" or "pre-term birth*" or "premature birth*" or prematur*) AND ((pregnan* or antenatal or "ante-natal" or prenatal))) OR ((tocolytic* or tocolysis*or betamimetic* or "beta agonist" or terbutaline or ritodrine or isoxsuprine or fenoterol or hexoprenaline or salbutamol or albuterol or "calcium channel blocker*" or nifedipine or nicardipine or verapamil or "cyclo-oxygenase inhibitor*" or "cox inhibitor*" or "cox-2 inhibitor*" or indomethacin or nimesulide or sulindac or ketorolac or rofecoxib or celecoxib or naproxen or "magnesium sulphate" or "magnesium sulfate" or mgso4 or "magnesium gluconate*" or "epsom salt*" or oxytocin "receptor antagonist*" or atosiban or barusiban or "nitric oxide donor*" or nitroglycerin or "isosorbide dinitrate" or "progestational agent*" or progesterone or relaxin) AND ("preterm birth*" or "pre-term birth*" or "premature birth*" or prematur*) AND ((pregnan* or antenatal or "ante-natal" or prenatal))) OR (("adrenal cortex hormone*" or corticosteroid* or corticoid* or glucocorticoid* or steroid* or betamethasone or dexamethasone or hydrocortisone) AND ("preterm birth*" or "pre-term birth*" or "premature birth*" or prematur*) AND ((pregnan* or antenatal or "ante-natal" or prenatal)))
