# Supplementary material for: Factors influencing appropriate use of interventions for management of women experiencing preterm birth: A mixed-methods systematic review and narrative synthesis
Source: PLoS Med. 2022 Aug 23;19(8):e1004074. doi: 10.1371/journal.pmed.1004074 (PMC9398034; doi:10.1371/journal.pmed.1004074)
Supplement: S4 Appendix — (PDF) [file pmed.1004074.s004.pdf]

S4 Appendix. Critical Appraisal

S4.1 Appendix – Critical appraisal of quantitative studies

Study details	Screening questions	2. Randomized controlled trials questions						4. Quantitative descriptive studies questions					
**Author**	**S1. Are there clear research questions?**	**S2. Do the collected data allow to address the research questions?**	**2.1. Is randomization appropriately performed?**	**2.2. Are the groups comparable at baseline?**	**2.3. Are there complete outcome data?**	**2.4. Are outcome assessors blinded to the intervention provided?**	**2.5 Did the participants adhere to the assigned intervention?**	**4.1. Is the sampling strategy relevant to address the research question?**	**4.2. Is the sample representative of the target population?**	**4.3. Are the measurements appropriate?**	**4.4. Is the risk of nonresponse bias low?**	**4.5. Is the statistical analysis appropriate to answer the research question?**	**MMAT Rating**
**Tucker Edmonds 2015 [1]**	Yes	Yes	N/A	N/A	N/A	N/A	N/A	Partial	No	Partial	Unclear	Yes	"Very low" (significant flaws impacting credibility/validity)
**Vargas-Origel 2000 [2]**	Yes	Yes	N/A	N/A	N/A	N/A	N/A	Yes	Partial	Partial	Unclear	Unclear	"Low" (some flaws likely to impact credibility/validity)
**Buchanan 2004 [3]**	Yes	Yes	N/A	N/A	N/A	N/A	N/A	Yes	Yes	Yes	Yes	Yes	"High" (no significant flaws)
**Bousleiman 2015 [4]**	Yes	Yes	N/A	N/A	N/A	N/A	N/A	Yes	Yes	Yes	Yes	Yes	"High" (no significant flaws)
**Battarbee 2020 [5]**	Yes	Yes	N/A	N/A	N/A	N/A	N/A	Yes	Unclear	Partial	Unclear	Yes	"Low" (some flaws likely to impact credibility/validity)
**Chan 2006 [6]**	Yes	Yes	N/A	N/A	N/A	N/A	N/A	Yes	Yes	Yes	Partial	Yes	"Moderate" (minor flaws impacting credibility/validity)
**Capeless 1987 [7]**	Yes	Yes	N/A	N/A	N/A	N/A	N/A	Yes	Yes	Unclear	Yes	Unclear	"Low" (some flaws likely to impact credibility/validity)
**Bain 2013 [8]**	Yes	Yes	N/A	N/A	N/A	N/A	N/A	Yes	Yes	Partial	Yes	Yes	"Moderate" (minor flaws

													impacting credibility/validity)
**Hueston 1997 [9]**	Yes	Yes	N/A	N/A	N/A	N/A	N/A	Yes	Yes	Yes	Yes	Yes	"High" (no significant flaws)
**Smith 2011 [10]**	Yes	Yes	N/A	N/A	N/A	N/A	N/A	Yes	Yes	Yes	Yes	Yes	"High" (no significant flaws)
**Battarbee 2019 [11]**	Yes	Yes	N/A	N/A	N/A	N/A	N/A	Yes	Yes	Yes	Yes	Yes	"High" (no significant flaws)
**Danerek 2012 [12]**	Yes	Yes	N/A	N/A	N/A	N/A	N/A	Yes	Yes	Yes	Yes	Yes	"High" (no significant flaws)
**Hutton 1989 [13]**	Yes	Yes	N/A	N/A	N/A	N/A	N/A	Yes	Yes	Unclear	Yes	Unclear	"Low" (some flaws likely to impact credibility/validity)
**Erickson 2001 [14]**	Yes	Yes	N/A	N/A	N/A	N/A	N/A	Yes	Yes	Partial	Partial	Yes	"Low" (some flaws likely to impact credibility/validity)
**Cook 2004 [15]**	Yes	Yes	N/A	N/A	N/A	N/A	N/A	Yes	Yes	Unclear	Partial	Yes	"Low" (some flaws likely to impact credibility/validity)
**Gatman 2020 [16]**	Yes	Yes	N/A	N/A	N/A	N/A	N/A	Yes	Yes	Yes	Yes	Yes	"High" (no significant flaws)
**Glass 2005 [17]**	Yes	Yes	N/A	N/A	N/A	N/A	N/A	Yes	Yes	Partial	Partial	Yes	"Moderate" (minor flaws impacting credibility/validity)
**Chollat 2017 [18]**	Yes	Yes	N/A	N/A	N/A	N/A	N/A	Yes	Yes	Unclear	Yes	Yes	"Moderate" (minor flaws impacting credibility/validity)
**Aleman 2013 [19]**	Yes	Yes	N/A	N/A	N/A	N/A	N/A	Yes	Yes	Yes	Partial	Yes	"Moderate" (minor flaws impacting credibility/validity)
**Baker 2015 [20]**	Yes	Yes	N/A	N/A	N/A	N/A	N/A	Yes	Yes	Unclear	Yes	Unclear	"Low" (some flaws likely to impact credibility/validity)
**Saengwaree 2005 [21]**	Yes	Yes	N/A	N/A	N/A	N/A	N/A	Yes	Yes	Partial	Unclear	Yes	"Low" (some flaws likely to impact credibility/validity)

**Wilson 2002 [22]**	Yes	Yes	N/A	N/A	N/A	N/A	N/A	Yes	Yes	Unclear	Unclear	Yes	“Low” (some flaws likely to impact credibility/validity)
**Hong 2017 [23]**	Yes	Yes	N/A	N/A	N/A	N/A	N/A	Yes	Yes	Unclear	Unclear	Yes	“Low” (some flaws likely to impact credibility/validity)
**Hui 2007 [24]**	Yes	Yes	N/A	N/A	N/A	N/A	N/A	Yes	Yes	Yes	No	Yes	“Moderate” (minor flaws impacting credibility/validity)
**Kenyon 2010 [25]**	Yes	Yes	N/A	N/A	N/A	N/A	N/A	Yes	Yes	Unclear	Yes	Unclear	“Low” (some flaws likely to impact credibility/validity)
**McGoldrick 2017 [26]**	Yes	Yes	N/A	N/A	N/A	N/A	N/A	Yes	Yes	Partial	Yes	Partial	“Moderate” (minor flaws impacting credibility/validity)
**Tucker Edmonds 2015 [27]**	Yes	Yes	N/A	N/A	N/A	N/A	N/A	Yes	Yes	Partial	Yes	Yes	“Moderate” (minor flaws impacting credibility/validity)
**Rousseau 2020 [28]**	Yes	Yes	N/A	N/A	N/A	N/A	N/A	Yes	Yes	Yes	No	Yes	“Moderate” (minor flaws impacting credibility/validity)
**Tucker Edmonds 2015 [29]**	Yes	Yes	N/A	N/A	N/A	N/A	N/A	Unclear	Unclear	Partial	Yes	Partial	“Low” (some flaws likely to impact credibility/validity)
**Liu 2015 [30]**	Yes	Yes	N/A	N/A	N/A	N/A	N/A	Yes	Unclear	Yes	Unclear	Yes	“Moderate” (minor flaws impacting credibility/validity)
**Aghajafari 2002 [31]**	Yes	Yes	Yes	Yes	Yes	Unclear	Yes	N/A	N/A	N/A	N/A	N/A	“Moderate” (minor flaws impacting credibility/validity)
**Kankaria 2021 [32]**	N/A	N/A	N/A	N/A	N/A	N/A	N/A	Yes	Partial	Yes	Yes	Yes	“Moderate” (minor flaws impacting credibility/validity)

S4.2 Appendix – Critical appraisal of qualitative studies

Study details	Screening questions		1. Qualitative study questions							
**Author**	**S1. Are there clear research questions?**	**S2. Do the collected data allow to address the research questions?**	**1.1. Is the qualitative approach appropriate to answer the research question? (Aim, appropriateness of a qualitative approach)**	**1.2. Are the qualitative data collection methods adequate to address the research question? (recruitment, data collection)**	**1.3. Are the findings adequately derived from the data? (rigor in analysis)**	**1.4. Is the interpretation of results sufficiently substantiated by data? (link from data to findings)**	**1.5. Is there coherence between qualitative data sources, collection, analysis and interpretation? (overall design from start to finish)**	**1.6. Have ethical issues been taken into consideration? (consent, confidentiality, ethics approval)**	**1.7. Is relationship between researcher and participants adequately considered? (interaction and reflection on how research team influences design & implementation)**	**MMAT RATING**
**Leviton 1995 [33]**	Yes	Yes	Yes	Partial	Partial	Unclear	Partial	Unclear	No	“Very low” (significant flaws impacting credibility/validity)
**McGoldrick 2016 [34]**	Yes	Yes	Yes	Partial	Yes	Yes	Yes	Yes	No	“Moderate” (minor flaws impacting credibility/validity)
**McGoldrick 2016 [35]**	Yes	Yes	Yes	Partial	Yes	Partial	Yes	Yes	No	“Moderate” (minor flaws impacting credibility/validity)
**Antony 2019 [36]**	Yes	Yes	Yes	Partial	Yes	Yes	Yes	Yes	No	“Moderate” (minor flaws impacting credibility/validity)
**Bain 2015 [37]**	Yes	Yes	Yes	Partial	Yes	Yes	Yes	Yes	Yes	"High” (no significant flaws)
**Hsieh 2006 [38]**	Yes	Yes	Yes	Yes	Yes	Yes	Yes	Yes	No	"High” (no significant flaws)
**Kaplan 2016 [39]**	Yes	Yes	Yes	Yes	Yes	Yes	Yes	Partial	Yes	“Moderate” (minor flaws impacting credibility/validity)
**Levison 2014 [40]**	Yes	Yes	Yes	Yes	Partial	Partial	Yes	Yes	No	“Low” (some flaws likely to impact credibility/validity)
**Greensides 2018 [41]**	Partial	Yes	Unclear	Yes	Unclear	Unclear	Partial	Unclear	No	“Very low” (significant flaws impacting credibility/validity)
**Kalb 1993 [42]**	Yes	Yes	Yes	Yes	Yes	Yes	Yes	Yes	Yes	"High” (no significant flaws)
**Hu 2006 [43]**	Yes	Yes	Yes	Partial	Yes	Yes	Partial	Unclear	No	“Low” (some flaws likely to impact credibility/validity)

S4.3 Appendix – Critical appraisal of mixed methods studies

Study details	Author	Smith 2016 [44]	Burhouse 2017 [45]	Teela 2015 [46]
**Screening questions**	S1. Are there clear research questions?	Yes	Yes	Yes
	S2. Do the collected data allow to address the research questions?	Yes	Yes	Yes
**1. Qualitative studies questions**	1.1. Is the qualitative approach appropriate to answer the research question? (Aim, appropriateness of a qualitative approach)	No	Yes	Yes
	1.2. Are the qualitative data collection methods adequate to address the research question? (recruitment, data collection)	Unclear	Yes	Partial
	1.3. Are the findings adequately derived from the data? (rigor in analysis)	No	Unclear	Yes
	1.4. Is the interpretation of results sufficiently substantiated by data? (link from data to findings)	Unclear	Unclear	Yes
	1.5. Is there coherence between qualitative data sources, collection, analysis, and interpretation? (overall design from start to finish)	Yes	Yes	Partial
	1.6. Have ethical issues been taken into consideration? (consent, confidentiality, ethics approval)	Yes	Partial	Partial
	1.7. Is relationship between researcher and participants adequately considered? (interaction and reflection on how research team influences design & implementation)	No	No	Partial
**3. Non-randomized studies questions**	3.1. Are the participants representative of the target population?	Yes	N/A	N/A
	3.2. Are measurements appropriate regarding both the outcome and intervention (or exposure)?	Partial	N/A	N/A
	3.3. Are there complete outcome data?	Partial	N/A	N/A
	3.4. Are the confounders accounted for in the design and analysis?	No	N/A	N/A
	3.5. During the study period, is the intervention administered (or exposure occurred) as intended?	Unclear	N/A	N/A
**4. Quantitative descriptive studies questions**	4.1. Is the sampling strategy relevant to address the research question?	N/A	Yes	Unclear
	4.2. Is the sample representative of the target population?	N/A	Yes	Unclear
	4.3. Are the measurements appropriate?	N/A	Yes	Partial
	4.4. Is the risk of nonresponse bias low?	N/A	Yes	Unclear
	4.5. Is the statistical analysis appropriate to answer the research question?	N/A	Yes	Unclear
**5. Mixed methods studies questions**	5.1. Is there an adequate rationale for using a mixed methods design to address the research question?	No	Partial	No
	5.2. Are the different components of the study effectively integrated to answer the research question?	Partial	Yes	Partial
	5.3. Are the outputs of the integration of qualitative and quantitative components adequately interpreted?	No	Yes	Partial
	5.4. Are divergences and inconsistencies between quantitative and qualitative results adequately addressed?	Yes	Unclear	No
	5.5. Do the different components of the study adhere to the quality criteria of each tradition of the methods involved?	Partial	Partial	Partial
**MMAT Rating**		"Very low" (significant flaws impacting credibility/validity)	"Low" (some flaws likely to impact credibility/validity)	"Very low" (significant flaws impacting credibility/validity)

References

[1] Edmonds BT, McKenzie F, Hendrix KS, Perkins SM, Zimet GD. The influence of resuscitation preferences on obstetrical management of periviable deliveries. J Perinatol 2015;35:161–6. https://doi.org/10.1038/jp.2014.175.

[2] Vargas-Origel A, León Ramírez D, Zamora-Orozco J, Vargas-Nieto MA. [Prenatal corticosteroids. Use and attitudes of the gynecology-obstetrics medical staff]. Ginecol Obstet Mex 2000;68:291–5.

[3] Buchanan S, Crowther C, Morris J. Preterm prelabour rupture of the membranes: a survey of current practice. Aust N Z J Obstet Gynaecol 2004;44:400–3. https://doi.org/10.1111/j.1479-828X.2004.00256.x.

[4] Bousleiman SZ, Rice MM, Moss J, Todd A, Rincon M, Mallett G, et al. Use and attitudes of obstetricians toward 3 high-risk interventions in MFMU Network hospitals. Am J Obstet Gynecol 2015;213:398.e1-11. https://doi.org/10.1016/j.ajog.2015.05.005.

[5] Battarbee AN, Aliaga S, Boggess KA. Management of diabetic women with threatened preterm birth: a survey of Maternal-Fetal Medicine providers. J Matern Fetal Neonatal Med 2020;33:2941–9. https://doi.org/10.1080/14767058.2019.1566307.

[6] Chan KL, Kean LH, Marlow N. Staff views on the management of the extremely preterm infant. Eur J Obstet Gynecol Reprod Biol 2006;128:142–7. https://doi.org/10.1016/j.ejogrb.2006.01.012.

[7] Capeless EL, Mead PB. Management of preterm premature rupture of membranes: lack of a national consensus. Am J Obstet Gynecol 1987;157:11–2. https://doi.org/10.1016/s0002-9378(87)80335-6.

[8] Bain E, Bubner T, Ashwood P, Crowther CA, Middleton P, WISH Project Team. Implementation of a clinical practice guideline for antenatal magnesium sulphate for neuroprotection in Australia and New Zealand. Aust N Z J Obstet Gynaecol 2013;53:86–9. https://doi.org/10.1111/ajo.12008.

[9] Hueston WJ. Variations between family physicians and obstetricians in the evaluation and treatment of preterm labor. J Fam Pract 1997;45:336–40.

[10] Smith V, Devane D, Higgins S. Practices for predicting and preventing preterm birth in Ireland: a national survey. Ir J Med Sci 2011;180:63–7. https://doi.org/10.1007/s11845-010-0604-1.

[11] Battarbee AN, Clapp MA, Boggess KA, Kaimal A, Snead C, Schulkin J, et al. Practice Variation in Antenatal Steroid Administration for Anticipated Late Preterm Birth: A Physician Survey. Am J Perinatol 2019;36:200–4. https://doi.org/10.1055/s-0038-1667028.

[12] Danerek M, Maršál K, Cuttini M, Lingman G, Nilstun T, Dykes A-K. Attitudes of Swedish midwives towards management of extremely preterm labour and birth. Midwifery 2012;28:e857-864. https://doi.org/10.1016/j.midw.2011.10.009.

[13] Hutton JD, Kee DG, Wilcox FL. New Zealand obstetricians' management of hypertension in pregnancy. A questionnaire survey. Aust N Z J Obstet Gynaecol 1989;29:5–8. https://doi.org/10.1111/j.1479-828x.1989.tb02866.x.

[14] Erickson K, Schmidt L, Santesso DL, Schulkin J, Gregory K, Hobel C. Obstetrician-gynecologists' knowledge and training about antenatal corticosteroids. Obstet Gynecol 2001;97:140–6. https://doi.org/10.1016/s0029-7844(00)01122-4.

[15] Cook C-M, Peek MJ. Survey of the management of preterm labour in Australia and New Zealand in 2002. Aust N Z J Obstet Gynaecol 2004;44:35–8. https://doi.org/10.1111/j.1479-828X.2004.00173.x.

[16] Gatman K, May R, Crowther C. Survey on use of antenatal magnesium sulphate for fetal neuroprotection prior to preterm birth in Australia and New Zealand - Ongoing barriers and enablers. Aust N Z J Obstet Gynaecol 2020;60:44–8. https://doi.org/10.1111/ajo.12981.

[17] Glass NE, Schulkin J, Chamany S, Riley LE, Schuchat A, Schrag S. Opportunities to reduce overuse of antibiotics for perinatal group B streptococcal disease prevention and management of preterm premature rupture of membranes. Infect Dis Obstet Gynecol 2005;13:5–10. https://doi.org/10.1080/10647440400028144.
[18] Chollat C, Le Doussal L, de la Villéon G, Provost D, Marret S. Antenatal magnesium sulphate administration for fetal neuroprotection: a French national survey. BMC Pregnancy Childbirth 2017;17:304. https://doi.org/10.1186/s12884-017-1489-z.
[19] Aleman A, Cafferata ML, Gibbons L, Althabe F, Ortiz J, Sandoval X, et al. Use of antenatal corticosteroids for preterm birth in Latin America: providers knowledge, attitudes and practices. Reprod Health 2013;10:4. https://doi.org/10.1186/1742-4755-10-4.
[20] Baker E, Hunter T, Okun N, Farine D. Current practices in the prediction and prevention of preterm birth in patients with higher-order multiple gestations. Am J Obstet Gynecol 2015;212:671.e1-671.e7. https://doi.org/10.1016/j.ajog.2014.12.031.
[21] Saengwaree P. Changing Physician’s Practice on Antenatal Corticosteroids in Preterm Birth. J Med Assoc Thai 2005;88:307–13.
[22] Wilson B, Thornton JG, Hewison J, Lilford RJ, Watt I, Braunholtz D, et al. The Leeds University Maternity Audit Project. Int J Qual Health Care 2002;14:175–81. https://doi.org/10.1093/oxfordjournals.intqhc.a002609.
[23] Hong JAX, Mathur M. Resident Quality Improvement Project: Antenatal Magnesium Sulfate Protocol for Fetal Neuroprotection in Preterm Births. Obstet Gynecol Int J 2017;Volume 7. https://doi.org/10.15406/ogij.2017.07.00265.
[24] Hui D, Liu G, Kavuma E, Hewson SA, McKay D, Hannah ME. Preterm labour and birth: a survey of clinical practice regarding use of tocolytics, antenatal corticosteroids, and progesterone. J Obstet Gynaecol Can 2007;29:117–24. https://doi.org/10.1016/S1701-2163(16)32384-2.
[25] Kenyon S, Pike K, Jones D, Brocklehurst P, Marlow N, Salt A, et al. Has publication of the results of the ORACLE Children Study changed practice in the UK? BJOG 2010;117:1344–9. https://doi.org/10.1111/j.1471-0528.2010.02661.x.
[26] McGoldrick EL, Brown JA, Groom KM, Crowther CA. Investigating antenatal corticosteroid clinical guideline practice at an organisational level. Aust N Z J Obstet Gynaecol 2017;57:25–32. https://doi.org/10.1111/ajo.12564.
[27] Tucker Edmonds B, McKenzie F, Farrow V, Raglan G, Schulkin J. A national survey of obstetricians’ attitudes toward and practice of periviable intervention. J Perinatol 2015;35:338–43. https://doi.org/10.1038/jp.2014.201.
[28] Rousseau A, Azria E, Baumann S, Deneux-Tharaux C, Senat MV. Do obstetricians apply the national guidelines? A vignette-based study assessing practices for the prevention of preterm birth. BJOG 2020;127:467–76. https://doi.org/10.1111/1471-0528.16039.
[29] Tucker Edmonds B, McKenzie F, Panoch JE, Barnato AE, Frankel RM. Comparing obstetricians’ and neonatologists’ approaches to periviable counseling. J Perinatol 2015;35:344–8. https://doi.org/10.1038/jp.2014.213.
[30] Liu G, Segrè J, Gülmezoglu AM, Mathai M, Smith JM, Hermida J, et al. Antenatal corticosteroids for management of preterm birth: a multi-country analysis of health system bottlenecks and potential solutions. BMC Pregnancy Childbirth 2015;15:S3. https://doi.org/10.1186/1471-2393-15-S2-S3.
[31] Aghajafari F, Murphy K, Ohlsson A, Amankwah K, Matthews S, Hannah ME. Multiple versus single courses of antenatal corticosteroids for preterm birth: a pilot study. J Obstet Gynaecol Can 2002;24:321–9. https://doi.org/10.1016/s1701-2163(16)30625-9.
[32] Kankaria A, Duggal M, Chauhan A, Sarkar D, Dalpath S, Kumar A, et al. Readiness to Provide Antenatal Corticosteroids for Threatened Preterm Birth in Public Health Facilities in Northern India. Glob Health Sci Pract 2021;9:575–89. https://doi.org/10.9745/GHSP-D-20-00716.
[33] Leviton LC, Baker S, Hassol A, Goldenberg RL. An exploration of opinion and practice patterns affecting low use of antenatal corticosteroids. Am J Obstet Gynecol 1995;173:312–6. https://doi.org/10.1016/0002-9378(95)90220-1.

[34] McGoldrick EL, Crawford T, Brown JA, Groom KM, Crowther CA. Consumers attitudes and beliefs towards the receipt of antenatal corticosteroids and use of clinical practice guidelines. BMC Pregnancy Childbirth 2016;16:259. https://doi.org/10.1186/s12884-016-1043-4.
[35] Mc Goldrick EL, Crawford T, Brown JA, Groom KM, Crowther CA. Identifying the barriers and enablers in the implementation of the New Zealand and Australian Antenatal Corticosteroid Clinical Practice Guidelines. BMC Health Serv Res 2016;16:617. https://doi.org/10.1186/s12913-016-1858-8.
[36] Antony KM, Levison J, Suter MA, Raine S, Chiudzu G, Phiri H, et al. Qualitative assessment of knowledge transfer regarding preterm birth in Malawi following the implementation of targeted health messages over 3 years. Int J Womens Health 2019;11:75–95. https://doi.org/10.2147/IJWH.S185199.
[37] Bain E, Bubner T, Ashwood P, Van Ryswyk E, Simmonds L, Reid S, et al. Barriers and enablers to implementing antenatal magnesium sulphate for fetal neuroprotection guidelines: a study using the theoretical domains framework. BMC Pregnancy Childbirth 2015;15:176. https://doi.org/10.1186/s12884-015-0618-9.
[38] Hsieh Y-H, Kao C-H, Gau M-L. The lived experience of first-time expectant fathers whose spouses are tocolyzed in hospital. J Nurs Res 2006;14:65–74. https://doi.org/10.1097/01.jnr.0000387563.49565.22.
[39] Kaplan HC, Sherman SN, Cleveland C, Goldenhar LM, Lannon CM, Bailit JL. Reliable implementation of evidence: a qualitative study of antenatal corticosteroid administration in Ohio hospitals. BMJ Qual Saf 2016;25:173–81. https://doi.org/10.1136/bmjqs-2015-003984.
[40] Levison J, Nanthuru D, Chiudzu G, Kazembe PN, Phiri H, Ramin SM, et al. Qualitative assessment of attitudes and knowledge on preterm birth in Malawi and within country framework of care. BMC Pregnancy Childbirth 2014;14:123. https://doi.org/10.1186/1471-2393-14-123.
[41] Greensides D, Robb-McCord J, Noriega A, Litch JA. Antenatal Corticosteroids for Women at Risk of Imminent Preterm Birth in 7 sub-Saharan African Countries: A Policy and Implementation Landscape Analysis. Glob Health Sci Pract 2018;6:644–56. https://doi.org/10.9745/GHSP-D-18-00171.
[42] Kalb KA. Women's experiences using terbutaline pump therapy for the management of preterm labor. PhD Dissertation. University of Minnesota, 1993.
[43] Hu Y-L. [Study of stress and coping behaviors in families of hospitalized pregnant woman undergoing tocolysis]. Hu Li Za Zhi 2006;53:45–52.
[44] Smith JM, Gupta S, Williams E, Brickson K, Ly Sotha K, Tep N, et al. Providing antenatal corticosteroids for preterm birth: a quality improvement initiative in Cambodia and the Philippines. Int J Qual Health Care 2016;28:682–8. https://doi.org/10.1093/intqhc/mzw095.
[45] Burhouse A, Lea C, Ray S, Bailey H, Davies R, Harding H, et al. Preventing cerebral palsy in preterm labour: a multiorganisational quality improvement approach to the adoption and spread of magnesium sulphate for neuroprotection. BMJ Open Qual 2017;6:e000189. https://doi.org/10.1136/bmjoq-2017-000189.
[46] Teela KC, De Silva DA, Chapman K, Synnes AR, Sawchuck D, Basso M, et al. Magnesium sulphate for fetal neuroprotection: benefits and challenges of a systematic knowledge translation project in Canada. BMC Pregnancy Childbirth 2015;15:347. https://doi.org/10.1186/s12884-015-0785-8.
