# Supplementary material for: Factors influencing appropriate use of interventions for management of women experiencing preterm birth: A mixed-methods systematic review and narrative synthesis
Source: PLoS Med. 2022 Aug 23;19(8):e1004074. doi: 10.1371/journal.pmed.1004074 (PMC9398034; doi:10.1371/journal.pmed.1004074)
Supplement: S5 Appendix — (PDF) [file pmed.1004074.s005.pdf]

S5 Appendix. GRADE-CERQual Evidence Profile

#	Summary of qualitative review findings	Contributing qualitative studies	Methodological limitations	Coherence	Relevance	Adequacy	Overall CERQual assessment	Explanation of overall assessment
**1**	**Inaccurate assessment of gestational age**							
**1.1**	**Limitations about determining gestational age**<br>Women and health providers reported that last menstrual period or last month of menstrual period were the most common methods in assessing gestational age in LMICs, despite health providers acknowledging their limited accuracy. Some health providers in these settings were aware of ultrasound assessments of gestational age, whereas community health workers were not aware on the role of ultrasound dating in pregnancy.	[1–3]	Moderate concerns: 1 study with minor issues (recruitment/data collection, reflexivity), 1 study with some issues (analysis, link from data to findings, reflexivity), and 1 study with substantial issues (appropriateness of qualitative approach, recruitment/data collection, analysis, link from data to findings, reflexivity).	No or very minor concerns	Moderate concerns: All studies indirectly relevant to review aims and represented 3 countries (Malawi, Cambodia, Philippines), including 2 low-income countries and 1 lower middle-income country. All perspectives came from health providers, women, and partners.	Minor concerns: 3 out of 14 studies contributed to review finding (2 thick and 1 thin data).	**Moderate confidence**	Due to no or very minor concerns regarding coherence, minor concerns regarding adequacy (2 of 14 studies with thick data, and 1 of 14 studies with thin data), moderate concerns regarding methodological limitations (appropriateness of qualitative approach, recruitment/data collection, analysis, link from data to findings, reflexivity), and relevance (all studies with indirect relevance).
**2**	**Inconsistent practice guidelines**							
**2.1**	**Inconsistent practice guidelines**<br>There were substantial variations in the content of practice and implementation guidelines on ACS across contexts, typically about appropriate gestational age criteria, determination of what constitutes imminence in preterm labour birth, how to exclude maternal infection that precludes ACS use, adequacy of childbirth and preterm newborn care environment, and use in specific populations of women.	[4,5]	Moderate concerns: 1 study with minor issues (recruitment/data collection, reflexivity), and 1 study with substantial issues (research aims, appropriateness of qualitative approach, analysis, link from data to findings, coherence on designs, ethics, reflexivity).	No or very minor concerns	No or very minor concerns: All studies directly relevant to review aims, and represented 9 countries (Democratic Republic of the Congo, Ethiopia, Malawi, Nigeria, Sierra Leone, Tanzania, Uganda, Australia, New Zealand),	Moderate concerns: 2 out 14 studies contributed to review finding (2 moderate to very thick data).	**Moderate confidence**	Due to no or very minor concerns regarding coherence and relevance, minor concerns regarding adequacy (2 of 14 studies with moderate to very thick data), moderate concerns regarding methodological limitations (recruitment/data collection, research aims, appropriateness of qualitative approach, analysis, link from data to findings, coherence on designs, ethics, reflexivity).

#	Summary of qualitative review findings	Contributing qualitative studies	Methodological limitations	Coherence	Relevance	Adequacy	Overall CERQual assessment	Explanation of overall assessment
					including 2 high-income countries and 7 low-income countries. All perspectives came from health providers.			
**3**	**Variable knowledge about the interventions**							
**3.1**	**Health providers' knowledge of the interventions** Health providers' knowledge about guidelines for and use of ACS, magnesium sulphate and tocolytics was variable. Where there was high levels of knowledge and experience in administering the interventions, this improved implementation feasibility. Lack of knowledge or outdated knowledge were barriers to appropriate use. The key existing knowledge gaps were related to differences between research evidence and previous clinical training or experience, which sometimes involved different courses, dosing, and duration.	[2–4,6–9]	Moderate concerns: 1 study with no or very minor issues, 3 studies with minor issues (recruitment/data collection, reflexivity, ethics), 1 study with some issues (analysis, link from data to findings, reflexivity), and 2 studies with substantial issues (recruitment/data collection, analysis, link from data to findings, coherence on designs, ethics, reflexivity).	No or very minor concerns	Moderate concerns: 4 studies directly and 3 studies indirectly relevant to review aim. 1 study published in 1995. Studies conducted in 5 countries (Australia, New Zealand, Malawi, United of States, Canada), including 4 high income countries and 1 low-income country. All perspectives came from health providers.	No or very minor concerns: 7 out 14 studies contributed to review finding (5 thick, 2 thin data).	**Moderate confidence**	Due to no or very minor concerns regarding coherence and adequacy, moderate concerns regarding methodological limitations (recruitment/data collection, analysis, link from data to findings, coherence on designs, ethics, reflexivity), and relevance (3 studies with indirect relevance)
**3.2**	**Knowledge about optimal gestational age for intervention administration** Knowledge about optimal gestational age for administration of ACS and magnesium sulphate varied across health providers, with mixed opinions about the earliest gestational age they would administer and agreement that these were challenging to	[4,5,8,9]	Serious concerns: 1 study with minor issues (recruitment/data collection, reflexivity), and 3 studies with substantial issues (research aims, appropriateness of qualitative research,	No or very minor concerns	Moderate concerns: 3 studies directly and 1 study indirectly relevant to review aim. 1 study published in 1995. Studies conducted in 11 countries (Australia, New	Minor concerns: 4 out of 14 studies contributed to review finding (3 moderate to very thick data and 1 thin data).	**Moderate confidence**	Due to no or very minor concerns regarding coherence, minor concerns regarding adequacy (3 of 14 studies with moderate to very thick data, and 1 of 14 studies with thin data), moderate concerns regarding relevance (1 study with indirect relevance), serious concerns regarding methodological

#	Summary of qualitative review findings	Contributing qualitative studies	Methodological limitations	Coherence	Relevance	Adequacy	Overall CERQual assessment	Explanation of overall assessment
	have with women and families. Opinion about optimal gestational age for administration of interventions were also balanced with other factors including estimated time to birth, threatened versus imminent preterm birth, and local standards of practice.		recruitment/data collection, analysis, link from data to findings, coherence on designs, ethics, reflexivity).		Zealand, United States of America, Canada, Democratic Republic of the Congo, Ethiopia, Malawi, Nigeria, Sierra Leone, Tanzania, and Uganda), including 4 high income countries and 7 low-income countries. All perspectives came from health providers.			limitations (research aims, appropriateness of qualitative research, recruitment/data collection, analysis, link from data to findings, coherence on designs, ethics, reflexivity).
**4**	**Perceived risks and benefits**							
**4.1**	**Uncertainties in prescribing and administering ACS for specific populations of women** Health providers had uncertainties and lacked confidence regarding certain aspects of prescribing and administering ACS, such as whether to use repeat doses, or whether to use ACS in specific clinical situations (such as in women with diabetes, hypertension, fetal complications, maternal infection, or PPROM).	[2,4,7]	Moderate concerns: 2 studies with minor issues (recruitment/data collection, reflexivity), and 1 study with substantial issues (recruitment/data collection, analysis, link from data to findings, coherence on designs, ethics, reflexivity).	No or very minor concerns	Moderate concerns: 2 studies directly and 1 study indirectly relevant to review aim. 1 study published in 1995. Studies conducted in 4 countries (Australia, New Zealand, United States of America, Malawi), including 3 high income countries and 1 low-income country. All perspectives came from health providers.	Minor concerns: 3 out 14 studies contributed to review finding (2 moderate to very thick data and 1 thin data).	**Moderate confidence**	Due to no or very minor concerns regarding coherence, minor concerns regarding adequacy (2 of 14 studies with moderate to very thick data, and 1 of 14 study with thin data), moderate concerns regarding methodological limitations (recruitment/data collection, analysis, link from data to findings, coherence on designs, ethics, reflexivity), and relevance (1 study with indirect relevance).

#	Summary of qualitative review findings	Contributing qualitative studies	Methodological limitations	Coherence	Relevance	Adequacy	Overall CERQual assessment	Explanation of overall assessment
**4.2**	**Scepticism of the evidence base for interventions** Health providers had mixed beliefs about the evidence supporting ACS and magnesium sulphate for fetal neuroprotection. While some providers agreed with and believed in the evidence supporting their use, others were sceptical about long-term outcomes, availability of high-quality trials, mixed evidence of effects and sufficiency of evidence, all of which may act as barriers to use.	[4,6–9]	Moderate concerns: 1 study with no or very minor issues, 2 studies with minor issues (recruitment/data collection, ethics, reflexivity), and 2 studies with substantial issues (recruitment/data collection, analysis, link from data to findings, coherence on designs, ethics, reflexivity).	No or very minor concerns	Moderate concerns: 4 studies directly and 1 study indirectly relevant to review aim. 1 study published in 1995. Studies conducted in 4 high income countries (Australia, New Zealand, Canada, United of States). All perspectives came from health providers.	Minor concerns: 5 out 14 studies contributed to review finding (4 thick, and 1 thin data).	**Moderate confidence**	Due to no or very minor concerns regarding coherence, minor concerns regarding adequacy (4 of 14 studies with thick data, and 1 of 14 studies with thin data), moderate concerns regarding methodological limitations (recruitment/data collection, analysis, link from data to findings, coherence on designs, ethics, reflexivity), and relevance (1 study with indirect relevance).
**4.3**	**Beliefs about risks of interventions** While many health providers believed that risks of ACS and magnesium sulphate were negligible, some had concerns about possible safety issues (particularly interactions with tocolytics, exacerbation of pulmonary oedema), low tolerance by women, long-term risks of complications for women, whether use at earlier gestational age is appropriate (<28 weeks), and risk of maternal infection.	[2,5,6,9]	Moderate concerns: 1 study with no or very minor issues, 1 study with minor issues (recruitment/data collection, reflexivity) and 2 studies with substantial issues (research aims, appropriateness of qualitative approach, recruitment/data collection, analysis, link from data to findings, coherence on designs, ethics, reflexivity).	No or very minor concerns	Moderate concerns: 3 studies directly and 1 study indirectly relevant to review aim. 1 study published in 1995. Studies conducted in 10 countries (Democratic Republic of the Congo, Ethiopia, Malawi, Nigeria, Sierra Leone, Tanzania, Uganda, Malawi, United States of America, Australia), including 2 high income countries and 8 low-income countries. All	Minor concerns: 4 out of 14 studies contributed to review finding (2 thick and 2 thin data).	**Moderate confidence**	Due to no or very minor concerns regarding coherence, minor concerns regarding adequacy (2 of 14 studies with thick data and 2 of 14 studies with thin data), moderate concerns regarding methodological limitations (research aims, appropriateness of qualitative approach, recruitment/data collection, analysis, link from data to findings, coherence on designs, ethics, reflexivity), and relevance (1 study with indirect relevance).

#	Summary of qualitative review findings	Contributing qualitative studies	Methodological limitations	Coherence	Relevance	Adequacy	Overall CERQual assessment	Explanation of overall assessment
					perspectives came from health providers.			
**4.4**	**Beliefs about risks of interventions – interaction with tocolytics** Some health providers believed that interaction of magnesium sulphate and ACS individually with tocolytic agents (particularly nifedipine) is associated with exacerbated adverse effects and toxicity for women. This belief may hinder administration of magnesium sulphate and ACS, in women who are also eligible for ACS and tocolytics.	[6,9]	Moderate concerns: 1study with no or very minor issues, and 1 study with substantial issues (recruitment/data collection, analysis, link from data to findings, coherence on designs, ethics, reflexivity).	No or very minor concerns	Moderate concerns: All studies directly relevant to review aims. 1 study published in 1995. Studies conducted in 2 high income countries (United States of America, Australia). All perspectives came from health providers.	Serious concerns: 2 out of 14 studies contributed to review finding (2 thin data).	**Low confidence**	Due to no or very minor concerns regarding coherence, moderate concerns regarding methodological limitations (recruitment/data collection, analysis, link from data to findings, coherence on designs, ethics, reflexivity), and relevance (1 study published in 1995 and all studies conducted in high income countries), serious concerns regarding adequacy (2 of 14 studies with thin data).
**4.5**	**Beliefs about benefits of interventions** Most health providers recognised the benefits of magnesium sulphate and ACS, believing that these interventions save lives, and benefits mostly outweigh risks. Women similarly believed that administration of ACS is beneficial, stressing the importance of using only when necessary and receiving information about potential side effects. In contrast, many health providers believed that tocolytics do not work and do not stop labour.	[2,4,6,7,9,10]	Minor concerns: 1 study with no or very minor issues, 4 studies with minor issues (recruitment/data collection, link from data to findings, ethics, reflexivity), and 1 study with substantial issues (recruitment/data collection, analysis, link from data to findings, coherence on designs, ethics, reflexivity).	No or very minor concerns	Moderate concerns: 5 studies directly and 1 study indirectly relevant to review aim. 1 study published in 1995. Studies conducted in 4 countries (United States of America, Australia, New Zealand, Malawi), including 3 high income countries and 1 low-income country. Perspectives came from health providers and women.	No or very minor concerns: 6 out 14 studies contributed to review finding (5 moderate to very thick data, 1 thin data).	**High confidence**	Due to no or very minor concerns regarding coherence and adequacy, minor concerns regarding methodological limitations (recruitment/data collection, analysis, link from data to findings, coherence on designs, ethics, reflexivity), moderate concerns regarding relevance (1 study with indirect relevance).
**5**	**Barriers in administration of interventions**							

#	Summary of qualitative review findings	Contributing qualitative studies	Methodological limitations	Coherence	Relevance	Adequacy	Overall CERQual assessment	Explanation of overall assessment
**5.1**	**Uncertainties on when to administer interventions** The unpredictability of preterm birth, including difficulty diagnosing threatened versus imminent preterm birth, can lead to provider hesitation in administering ACS and magnesium sulphate – providers fear being held responsible or blamed for potentially unnecessary treatment. To cope with these uncertainties, providers may delay treatment, preferring a “wait and see” approach.	[2,4,6,8,9]	Moderate concerns: 1 study with no or very minor issues, 2 study with minor issues (recruitment/data collection, reflexivity), and 2 studies with substantial issues (recruitment/data collection, analysis, link from data to findings, coherence on designs, ethics, reflexivity).	No or very minor concerns	Moderate concerns: 3 studies directly and 2 studies indirectly relevant to review aim. 1 study published in 1995. Studies conducted in 5 countries (Australia, New Zealand, United of States of America, Canada, Malawi), including 4 high income countries and 1 low-income country. All perspectives came from health providers.	Minor concerns: 5 out of 14 studies contributed to review finding (2 thick and 3 thin data).	**Moderate confidence**	Due to no or very minor concerns regarding coherence, minor concerns regarding adequacy (2 of 14 studies with thick data, and 3 of 14 studies with thin data), moderate concerns regarding methodological limitations (recruitment/data collection, analysis, link from data to findings, coherence on designs, ethics, reflexivity), and relevance (2 studies with indirect relevance).
**5.2**	**Time constraints and complexity in prescribing and administering** Health providers described time constraints in prescribing and administering ACS and magnesium sulphate as a critical overarching barrier to appropriate use, due to the acute nature and time pressures of imminent preterm birth, high intensity of workload, and competing tasks. Many health providers believed that prescribing and administering magnesium sulphate is complex, as preparation takes too much time, or is difficult to ‘draw it all up’, which could deter health providers in administering the	[4,6,8]	Moderate concerns: 1 study with no or very minor issues, 1 study with minor issues (recruitment/data collection, reflexivity), and 1 study with substantial issues (recruitment/data collection, coherence on findings, ethics, reflexivity).	No or very minor concerns	Moderate concerns: 2 studies directly and 1 study indirectly relevant to review aim. Studies conducted in 3 high income countries (Australia, New Zealand, Canada), All perspectives came from health providers.	Moderate concerns: 3 out of 14 studies contributed to review finding (1 thick and 2 thin data).	**Moderate confidence**	Due to no or very minor concerns regarding coherence, moderate concerns regarding methodological limitations (recruitment/data collection, coherence on findings, ethics, reflexivity), relevance (1 study with indirect relevance), and adequacy (1 of 14 studies with thick data, and 2 of 14 studies with thin data).

#	Summary of qualitative review findings	Contributing qualitative studies	Methodological limitations	Coherence	Relevance	Adequacy	Overall CERQual assessment	Explanation of overall assessment
	medication when they feel under pressure.							
**5.3**	**Stocking medications in maternity ward** Maintaining consistent stock of ACS and magnesium sulphate that is readily available in the maternity ward and emergency department, and the availability of health providers who are readily able to assess women in preterm labour, was critical to ensure that women received prompt treatment.	[1,2,5–7]	Moderate concerns: 1 study with no or very minor issues, 2 studies with minor issues (ethics, recruitment/data collection, reflexivity), and 2 studies with substantial issues (research aims, appropriateness of qualitative approach, recruitment/data collection, analysis, link from data to findings, coherence on designs, ethics, reflexivity).	No or very minor concerns	Minor concerns: 3 studies directly and 2 studies indirectly relevant to review aim. Studies conducted in 11 countries (United States of America, Australia, Democratic Republic of the Congo, Ethiopia, Malawi, Nigeria, Sierra Leone, Tanzania, Uganda, Cambodia, Philippines), including 2 high income countries, 9 low-income countries and 1 lower middle-income country. All perspectives came from health providers.	Minor concerns: 5 out 14 studies contributed to review finding (3 moderate to very thick data and 2 thin data).	**High confidence**	Due to no or very minor concerns regarding coherence, minor concerns regarding relevance (2 studies with indirect relevance), and adequacy (3 of 14 studies with moderate to very thick data, and 2 of 14 studies with thin data), moderate concerns regarding methodological limitations (research aims, appropriateness of qualitative approach, recruitment/data collection, analysis, link from data to findings, coherence on designs, ethic, reflexivity).
**5.4**	**Regulatory policies and beliefs about prescribing and administering authority** National-level guidance is often limited about who can prescribe and administer ACS and magnesium sulphate; where there is guidance, typically only obstetricians are authorized to prescribe and administer, while other health providers can	[4–7]	Minor concerns: 1 study with no or very minor issues, 2 studies with minor issues (recruitment/data collection, reflexivity, ethics), and 1 study with substantial issues (research aims,	No or very minor concerns	No or very minor concerns: All studies directly relevant to review aims and represented 10 countries (Democratic Republic of the Congo, Ethiopia, Malawi, Nigeria,	Minor concerns: 4 out of 14 studies contributed to review finding (all thick data).	**High confidence**	Due to no or very minor concerns regarding coherence and relevance, minor concerns understanding of the qualitative evidence as regarding methodological limitations (research aims, appropriateness of qualitative approach, analysis, link from data to findings, coherence on designs, ethics, reflexivity),

#	Summary of qualitative review findings	Contributing qualitative studies	Methodological limitations	Coherence	Relevance	Adequacy	Overall CERQual assessment	Explanation of overall assessment
	administer under clinical oversight, but not prescribe. Many health providers (obstetricians, neonatologists, midwives) likewise believe that prescription and administration of ACS and magnesium sulphate should be prescribed and administered by obstetricians-only, even though multi-disciplinary decision-making was highly valued.		appropriateness of qualitative approach, analysis, link from data to findings, coherence on designs, ethics, reflexivity).		Sierra Leone, Tanzania, and Uganda, Australia, New Zealand, Unites States of America), including 7 low-income countries and 3 high income countries. All perspectives came from health providers.			and adequacy (4 of 14 studies with thick data).
**6**	**Appropriate settings for administration**							
**6.1**	**Appropriate settings for ACS administration** In some national guidelines and in clinical practice, administration of ACS is allowed at only at tertiary facilities where comprehensive emergency obstetric and newborn care (CEmONC) and essential preterm newborn care interventions are available. While some country guidelines allow pre-referral first dose administration of ACS at lower-level facilities (where basic emergency obstetric and newborn care (BEmONC is available), implementation is limited due to challenges around identifying preterm labour, lack of knowledge about importance of pre-referral dosing, and transportation issues.	[2,3,5–8]	Moderate concerns: 6 studies, where 1 study with no or very minor issues, 2 studies with minor issues (recruitment/data collection, ethics, reflexivity), 1 study with some issues (analysis, link from data to findings, reflexivity), and 2 studies with substantial issues (research aims, appropriateness of qualitative approach, analysis, link from data to findings, coherence on designs, ethics, reflexivity, recruitment/data collection).	No or very minor concerns	Minor concerns: 3 studies directly and 3 studies indirectly relevant to review aim. Studies conducted in 10 countries (Democratic Republic of the Congo, Ethiopia, Malawi, Nigeria, Sierra Leone, Tanzania, Uganda, Australia, Unites States of America, Canada), including 7 low-income countries and 3 high income countries. All perspectives came from health providers.	No or very minor concerns: 6 out of 14 studies contributed to review finding (3 moderate to very thick data and 3 thin data).	**High confidence**	Due to no or very minor concerns regarding coherence and adequacy, minor concerns regarding relevance (3 studies with indirect relevance), moderate concerns regarding methodological limitations (research aims, appropriateness of qualitative approach, analysis, link from data to findings, coherence on designs, ethics, reflexivity, recruitment/data collection).

#	Summary of qualitative review findings	Contributing qualitative studies	Methodological limitations	Coherence	Relevance	Adequacy	Overall CERQual assessment	Explanation of overall assessment
7	**Strategies to improve appropriate use**							
**7.1**	**Implementing reminder systems and educational materials** Reminder systems and printed education materials (pamphlets, posters, signage) to prompt staff to prescribe and administer magnesium sulphate and ACS can facilitate appropriate use.	[4,6,7]	Minor concerns: 1 study with no or very minor issues, and 2 studies with minor issues (recruitment/data collection, ethics, reflexivity).	No or very minor concerns	No or very minor concerns: All studies directly relevant to review aims and represented3 high income countries (Australia, USA, New Zealand). All perspectives came from health providers. Not graded down due to absence in LMICs, as it does not mean reminder system are not important in LMICs and likely has not been trialled in LMICs. These three studies are implementation research study.	Minor concerns: 3 out of 14 studies contributed to review finding (2 thick and 1 thin data).	**High confidence**	Due to no or very minor concerns regarding coherence and relevance, minor concerns regarding methodological limitations (recruitment/data collection, ethics, reflexivity) and adequacy (2 of 14 studies with thick data and 1 of 14 studies with thin data)
**7.2**	**Developing reporting indicators and audit and feedback cycles** Developing and implementing key performance indicators on magnesium sulphate and ACS use for health facilities and implementing audit and feedback cycles may be enablers to encourage appropriate use.	[1,5–7]	Moderate concerns: 1 study with no or very minor issues, 1 study with minor issues (ethics), and 2 studies with substantial issues (research aims, appropriateness of qualitative approach, recruitment/data collection, analysis, link from data to	No or very minor concerns	Minor concerns: 3 studies directly and 1 study indirectly relevant to review aim. Studies conducted in 11 countries (Australia, Congo, Ethiopia, Malawi, Nigeria, Sierra Leone, Tanzania, Uganda, USA, Cambodia,	Minor concerns: 4 out of 14 studies contributed to review finding (2 thick and 2 thin data).	**Moderate confidence**	Due to no or very minor concerns regarding coherence, minor concerns regarding relevance (1 study with indirect relevance), and adequacy (2 of 14 studies with thick data and 2 of 14 studies with thin data), moderate concerns regarding methodological limitations (research aims, appropriateness of qualitative approach, recruitment/data collection, analysis, link from

#	Summary of qualitative review findings	Contributing qualitative studies	Methodological limitations	Coherence	Relevance	Adequacy	Overall CERQual assessment	Explanation of overall assessment
			findings, coherence on designs, ethics, reflexivity)		Philippines), including 2 high income countries and 9 low-income countries. All perspectives came from health providers and people on key organization.			data to findings, coherence on designs, ethics, reflexivity).
**7.3**	**Implementing education and training for health providers** Training for health providers to improve their knowledge about current research evidence, knowledge about impact of treatment on the woman and baby, and skills to administer ACS and magnesium sulphate were viewed as highly necessary and valuable.	[2,4–8,11]	Moderate concerns: 1 study with no or very minor issues, 3 studies with minor issues (recruitment/data collection, ethics, reflexivity), 1 study with some issues (analysis, link from data to findings, ethics, reflexivity), and 2 studies with substantial issues (research aims, appropriateness of qualitative approach, analysis, link from data to findings, coherence, ethics, reflexivity).	No or very minor concerns	Minor concerns: 4 studies directly and 3 studies indirectly relevant to review aim. Studies conducted in 13 countries (USA, Malawi, Australia, New Zealand, Congo, Ethiopia, Malawi, Nigeria, Sierra Leone, Tanzania, Uganda, UK, Canada), including 4 high income countries and 8 low-income countries. All perspectives came from health providers.	No or very minor concerns: 7 out 14 studies contributed to review finding (5 thick, and 2 thin data).	**High confidence**	Due to no or very minor concerns regarding coherence and adequacy, minor concerns regarding relevance (3 studies with indirect relevance), moderate concerns regarding methodological limitations (research aims, study design, analysis, link from data to findings, coherence, ethics, reflexivity).
**7.4**	**Appointing "change champions"** Nominating facility-level influential obstetricians and midwives as "change champions" may help to promote and enable magnesium sulphate and ACS training and use.	[6–8,11]	Moderate concerns: 1 study with no or very minor issues, 1 study with minor issues (ethics),1 study with some issues (rigor in analysis, link from	No or very minor concerns	Minor concerns: 2 studies directly and 2 studies indirectly relevant to review aim. Studies conducted in 4 high income	Minor concerns: 4 out 14 studies contributed to review finding (2 thick, 2 thin data).	**Moderate confidence**	Due to no or very minor concerns regarding coherence, minor concerns regarding relevance (2 studies with indirect relevance), and adequacy (2 of 14 studies with thick data and 2 of 14 studies with thin data), moderate

#	Summary of qualitative review findings	Contributing qualitative studies	Methodological limitations	Coherence	Relevance	Adequacy	Overall CERQual assessment	Explanation of overall assessment
			data to findings, ethics reflexivity), and 1 study with substantial issues (recruitment/data collection, coherence, ethics, reflexivity).		countries (Australia, USA, UK, Canada). All perspectives came from health providers. Not graded down due to absence in LMICs, as it does not mean change champion are not important in LMICs and likely has not been trialled in LMICs. These four studies are implementation research study.			concerns regarding methodological limitations (recruitment/data collection, ethics, rigor in analysis, link from data to findings, reflexivity, coherence).
**7.5**	**Multi-disciplinary teamwork to improve quality of care** Multi-disciplinary teamwork was highly valued by health providers to optimize ACS use, but fears, concerns and frustrations were expressed over poor communication between the obstetric, midwifery, neonatal and paediatric teams. Improved and standardized communication on ACS during handover and referral were highly valued but often lacking, particularly regarding whether interventions were administered yet and timing of administration.	[4,7]	Minor concerns: 2 studies with minor issues (recruitment/data collection, ethics, reflexivity)	No or very minor concerns	Minor concerns: All studies directly relevant to review aims and represented 3 high income countries (Australia, United States of America, New Zealand). All perspectives came from health providers.	Minor concerns: 2 out of 14 studies contributed to review finding (all thick data)	**High confidence**	Due to no or very minor concerns regarding coherence, minor concerns regarding methodological limitations (recruitment/data collection, ethics, reflexivity), relevance (3 studies conducted in high income countries), and adequacy (2 of 14 studies with thick data).
**8**	**Women's perspectives and experiences**							
**8.1**	**Women and partners' knowledge of interventions**	[2,3,10]	Moderate concerns: 2 studies with minor issues	No or very minor concerns	Minor concerns: 1 study directly and 2 studies	Moderate concerns: 3 out 14 studies	**Moderate confidence**	Due to no or very minor concerns regarding coherence, minor concerns

#	Summary of qualitative review findings	Contributing qualitative studies	Methodological limitations	Coherence	Relevance	Adequacy	Overall CERQual assessment	Explanation of overall assessment
	Women's and partners' knowledge of ACS varied across settings. In high-income countries, some women and partners understood that ACS improved fetal lung maturity, but were less aware of number of doses or the name of the medication administered. In contrast, in LMIC settings, very few women or their partners were aware of ACS.		(recruitment/data collection, reflexivity), and 1 study with some issues (analysis, link from data to findings, reflexivity).		indirectly relevant to review aim. Studies conducted in 3 countries (Australia, New Zealand, Malawi), including 2 high income countries, and 1 low-income country. Perspectives came from women and partners.	contributed to review finding (1 thick and 2 thin data).		regarding relevance (2 studies with indirect relevance), moderate concerns regarding methodological limitations (recruitment/data collection, reflexivity, analysis, link from data to findings), and adequacy (1 of 14 studies with thick data and 2 of 14 studies with thin data).
**8.2**	**Women learning about preterm birth management** Many women and partners first learned about preterm birth and its management (including use of tocolytics, ACS, magnesium sulphate) during emergency situations, hindering their understanding about potential interventions and sometimes contributing to hesitancy when risks and benefits were not well understood. Some women felt that decisions concerning ACS administration should be made solely by health providers, while others felt that they needed adequate time and information to consider risks and benefits. Women felt that their knowledge and ability to make informed decisions was improved by clear communication, adequate time for discussion with their provider, as well as educational sessions and materials.	[2,6,7,10,12–14]	Minor concerns: 3 studies with no or very minor issues, 3 studies with minor issues (recruitment/data collection, reflexivity, ethics, link from data to findings), and 1 study with some issues (coherence on designs, ethics, reflexivity).	No or very minor concerns	Moderate concerns: 6 studies directly and 1 study indirectly relevant to review aim. 1 study published in 1993. Studies conducted in 5 countries (Australia, Malawi, USA, Taiwan, New Zealand), including 4 high income countries and 1 low-income country. Perspectives came from health providers, women, and partners.	No or very minor concerns: 7 out of 14 studies contributed to review finding (all moderate to very thick data).	**High confidence**	Due to no or very minor concerns regarding coherence and adequacy, minor concerns regarding methodological limitations (recruitment/data collection, reflexivity, ethics, link from data to findings, coherence on designs), moderate concerns regarding relevance (1 study with indirect relevance).

#	Summary of qualitative review findings	Contributing qualitative studies	Methodological limitations	Coherence	Relevance	Adequacy	Overall CERQual assessment	Explanation of overall assessment
**8.3**	**Women's experiences of and concerns about side effects** Despite personal experiences of and concerns about potential side effects of tocolytics and ACS among women in high income countries, women mostly felt that they would take tocolytics and ACS in a future pregnancy if indicated. Some women preferred intravenous to oral tocolytics, as side effects were more consistent, with fewer "peaks and troughs" and uterine contractions.	[10,12,14]	Minor concerns: 2 studies with no or very minor issues, and 1 study with minor issues (recruitment/data collection, link from data to findings, reflexivity)	No or very minor concerns	Moderate concerns: All studies directly relevant to review aim. 1 study published in 1993. Studies conducted in 4 high income countries (Australia, USA, Taiwan, New Zealand). All perspectives came from women and partners.	Minor concerns: 3 out of 14 studies contributed to review finding (2 thick and 1 thin data).	**Moderate confidence**	Due to no or very minor concerns regarding coherence, minor concerns regarding methodological limitations (recruitment/data collection, link from data to findings, reflexivity), and adequacy (2 of 14 studies with thick data and 1 of 14 studies with thin data), moderate concerns regarding relevance (1 study published in 1993 and all studies conducted in high income countries)
**8.4**	**Women's concerns about on impact of interventions on baby** Women and partners expressed concerns about the baby's health – both from the possibility of preterm birth, and from the potential impact of tocolytics on the baby. Balancing the fear of these two unknowns could be highly stressful, particularly as some women described feeling decreased fetal movement after tocolytic administration.	[12–14]	Minor concerns: 2 studies with no or very minor issues, and 1 study with some issues (coherence on designs, ethics, reflexivity).	No or very minor concerns	Moderate concerns: All studies directly relevant to review aims. 1 study published in 1993. Studies conducted in 2 high income countries (United States of America, Taiwan). All perspectives came from women and partners.	Minor concerns: 3 out of 14 studies contributed to review finding (all thick data).	**Moderate confidence**	Due to no or very minor concerns regarding coherence, minor concerns regarding methodological limitations (coherence on designs, ethics, reflexivity), and adequacy (3 of 14 studies with thick data), moderate concerns regarding relevance (1 study published in 1993 and all studies conducted in high income countries)
**8.5**	**Regaining control and empowerment** Women experiencing preterm labour placed high value on interventions that helped them to maintain autonomy and regain control over their bodies and premature labour, such as interventions that enabled them to stay out of hospital or regain	[12]	No or very minor concerns: 1 study with no or very minor issues	No or very minor concerns	Moderate concerns: 1 study directly relevant to review aim, published in 1993, and represented 1 high income country (USA). All perspectives	Serious concerns: 1 out of 14 studies contributed to review finding (all thick data).	**Low confidence**	Due to no or very minor concerns regarding methodological limitations and coherence, moderate concerns regarding relevance (the study published in 1993 and conducted in high income country), serious concerns regarding adequacy (1 of 14 studies with thick data).

#	Summary of qualitative review findings	Contributing qualitative studies	Methodological limitations	Coherence	Relevance	Adequacy	Overall CERQual assessment	Explanation of overall assessment
	mobility. These types of interventions helped to promote their freedom while giving them a sense of security regarding their baby's health.				came from women.			
**8.6**	**Trust and relationships between women and health providers** Women highly valued time and space to have a two-way conversation and build trust with their health providers to understand their condition and treatment options. While some women reported experiencing positive relationships with health providers, critical threats to building trust included insufficient health provider time due to workload, lack of continuity of carers, and perceived invalidation of women's concerns about whether they were in labour or not.	[10,12,14]	Minor concerns: 2 studies with no or very minor issues, and 1 study with minor issues (recruitment/data collection, link from data to findings, reflexivity).	No or very minor concerns	Moderate concerns: All studies directly relevant to review aims. 1 study published in 1993. Studies conducted in 4 high income countries (USA, Australia, New Zealand, Taiwan). Perspectives came from women and partners.	Minor concerns: 3 out of 14 studies contributed to review finding (all moderate to very thick data).	**Moderate confidence**	Due to no or very minor concerns regarding coherence, minor concerns regarding methodological limitations (recruitment/data collection, link from data to findings, reflexivity), and adequacy (3 of 14 studies with moderate to very thick data), moderate concerns regarding relevance (1 study published in 1993, and all studies conducted in high income countries).
**8.7**	**Seeking support from families and peers** During preterm birth management, women leaned on their families and partners for emotional and physical support, such as motivation for staying on bedrest, general advice about pregnancy and baby health, sharing experiences, and developing coping strategies. Several women and their partners described it as challenging to ask for support from families and friends during preterm birth management, as it is less common to ask for support during	[10,12,14]	Minor concerns: 2 studies with no or very minor issues, and 1 study with minor issues (recruitment/data collection, link from data to findings, reflexivity).	No or very minor concerns	Moderate concerns: All studies directly relevant to review aims. 1 study published in 1993. Studies conducted in 4 high income countries (USA, Australia, New Zealand, Taiwan). Perspectives came from women and partners.	Minor concerns: 3 out of 14 studies contributed to review finding (all moderate to very thick data).	**Moderate confidence**	Due to no or very minor concerns regarding coherence, minor concerns regarding methodological limitations (recruitment/data collection, link from data to findings, reflexivity), and adequacy (3 of 14 studies with moderate to very thick data), moderate concerns regarding relevance (1 study published in 1993 and all studies conducted in high income countries).

#	Summary of qualitative review findings	Contributing qualitative studies	Methodological limitations	Coherence	Relevance	Adequacy	Overall CERQual assessment	Explanation of overall assessment
	pregnancy compared to after the baby is born.							
**8.8**	**Coping strategies – reframing experiences** For women and their partners, reframing experiences of preterm birth management was critical to avoid disappointment and strengthen resolve. Reframing experiences led women and their partners to attempt to focus on positive aspects of their lives, enjoying moments with the baby, building relationships with babies, and learning to let go.	[12,14]	No or very minor concerns: 2 studies with no or very minor issues.	No or very minor concerns	Moderate concerns: All studies directly relevant to review aim. 1 study published in 1993. Studies conducted in 2 high income countries (USA, Taiwan), Perspectives came from women and partners.	Minor concerns: 2 out of 14 studies contributed to review finding (all thick data).	**Moderate confidence**	Due to no or very minor concerns regarding methodological limitations and coherence, minor concerns regarding adequacy (2 of 14 studies with thick data), moderate concerns regarding relevance (1 study published in 1993 and all studies conducted in high income countries).

References

[1] Smith JM, Gupta S, Williams E, Brickson K, Ly Sotha K, Tep N, et al. Providing antenatal corticosteroids for preterm birth: a quality improvement initiative in Cambodia and the Philippines. Int J Qual Health Care 2016;28:682–8. https://doi.org/10.1093/intqhc/mzw095.

[2] Antony KM, Levison J, Suter MA, Raine S, Chiudzu G, Phiri H, et al. Qualitative assessment of knowledge transfer regarding preterm birth in Malawi following the implementation of targeted health messages over 3 years. Int J Womens Health 2019;11:75–95. https://doi.org/10.2147/IJWH.S185199.

[3] Levison J, Nanthuru D, Chiudzu G, Kazembe PN, Phiri H, Ramin SM, et al. Qualitative assessment of attitudes and knowledge on preterm birth in Malawi and within country framework of care. BMC Pregnancy Childbirth 2014;14:123. https://doi.org/10.1186/1471-2393-14-123.

[4] Mc Goldrick EL, Crawford T, Brown JA, Groom KM, Crowther CA. Identifying the barriers and enablers in the implementation of the New Zealand and Australian Antenatal Corticosteroid Clinical Practice Guidelines. BMC Health Serv Res 2016;16:617. https://doi.org/10.1186/s12913-016-1858-8.

[5] Greensides D, Robb-McCord J, Noriega A, Litch JA. Antenatal Corticosteroids for Women at Risk of Imminent Preterm Birth in 7 sub-Saharan African Countries: A Policy and Implementation Landscape Analysis. Glob Health Sci Pract 2018;6:644–56. https://doi.org/10.9745/GHSP-D-18-00171.

[6] Bain E, Bubner T, Ashwood P, Van Ryswyk E, Simmonds L, Reid S, et al. Barriers and enablers to implementing antenatal magnesium sulphate for fetal neuroprotection guidelines: a study using the theoretical domains framework. BMC Pregnancy Childbirth 2015;15:176. https://doi.org/10.1186/s12884-015-0618-9.

[7] Kaplan HC, Sherman SN, Cleveland C, Goldenhar LM, Lannon CM, Bailit JL. Reliable implementation of evidence: a qualitative study of antenatal corticosteroid administration in Ohio hospitals. BMJ Qual Saf 2016;25:173–81. https://doi.org/10.1136/bmjqs-2015-003984.

[8] Teela KC, De Silva DA, Chapman K, Synnes AR, Sawchuck D, Basso M, et al. Magnesium sulphate for fetal neuroprotection: benefits and challenges of a systematic knowledge translation project in Canada. BMC Pregnancy Childbirth 2015;15:347. https://doi.org/10.1186/s12884-015-0785-8.

[9] Leviton LC, Baker S, Hassol A, Goldenberg RL. An exploration of opinion and practice patterns affecting low use of antenatal corticosteroids. Am J Obstet Gynecol 1995;173:312–6. https://doi.org/10.1016/0002-9378(95)90220-1.

[10] McGoldrick EL, Crawford T, Brown JA, Groom KM, Crowther CA. Consumers attitudes and beliefs towards the receipt of antenatal corticosteroids and use of clinical practice guidelines. BMC Pregnancy Childbirth 2016;16:259. https://doi.org/10.1186/s12884-016-1043-4.

[11] Burhouse A, Lea C, Ray S, Bailey H, Davies R, Harding H, et al. Preventing cerebral palsy in preterm labour: a multiorganisational quality improvement approach to the adoption and spread of magnesium sulphate for neuroprotection. BMJ Open Qual 2017;6:e000189. https://doi.org/10.1136/bmjoq-2017-000189.

[12] Kalb KA. Women's experiences using terbutaline pump therapy for the management of preterm labor. PhD Dissertation. University of Minnesota, 1993.

[13] Hu Y-L. [Study of stress and coping behaviors in families of hospitalized pregnant woman undergoing tocolysis]. Hu Li Za Zhi 2006;53:45–52.

[14] Hsieh Y-H, Kao C-H, Gau M-L. The lived experience of first-time expectant fathers whose spouses are tocolyzed in hospital. J Nurs Res 2006;14:65–74. https://doi.org/10.1097/01.jnr.0000387563.49565.22.
