# Supplementary material for: Factors influencing appropriate use of interventions for management of women experiencing preterm birth: A mixed-methods systematic review and narrative synthesis
Source: PLoS Med. 2022 Aug 23;19(8):e1004074. doi: 10.1371/journal.pmed.1004074 (PMC9398034; doi:10.1371/journal.pmed.1004074)
Supplement: S6 Appendix — (PDF) [file pmed.1004074.s006.pdf]

S6 Appendix. Summary of Quantitative Findings

#	Summary of Quantitative review findings	Contributing quantitative studies	Quality ratings
**1**	**Inaccurate assessment of gestational age**		
**1.1**	**Limitations about determining gestational age**<br>Quantitative evidence extended the understanding of qualitative evidence that accurate and reliable gestational age assessments in LMICs was limited. Ultrasound gestational age dating was typically only available at higher level hospitals, which may hinder appropriate use of ACS. Similarly, to qualitative evidence, health providers routinely used fundal height, followed by last menstrual period and ultrasound.	[1]	1 study with moderate quality study
**2**	**Inconsistent practice guidelines**		
**2.1**	**Inconsistent practice guidelines**<br>Quantitative evidence supported the qualitative findings that the presence and content of guidelines or policy on ACS and magnesium sulphate varies across settings at national and health facility levels. Where country-level policy and guidelines for ACS existed, they were perceived as outdated, unclear, or were not widely disseminated.	[1–5]	From 5 studies (4 moderate and 1 low quality studies).
**3**	**Variable knowledge about the interventions**		
**3.1**	**Health providers' knowledge of the interventions**<br>Quantitative evidence supported the qualitative findings around variable knowledge on ACS. In India, health providers were reported to be confident in administering ACS, despite poor score on knowledge assessment regarding the intervention. Facilitators of ACS, magnesium sulphate and tocolytics use in relation to knowledge included health providers' positive attitudes, better knowledge, exposure to trainings, conferences, guidelines, and research articles. Barriers included lack of experience in administration, misinformation about correct use, and knowledge gaps on dosing and frequency.	[1,5–10]	From 13 studies (2 high, 5 moderate, 5 low, and 1 very low-quality studies)
**3.2**	**Knowledge about optimal gestational age for intervention administration**<br>Quantitative evidence supported the qualitative findings about health providers knowledge about the importance of gestational age for ACS and tocolytics administration, and that knowledge about optimal gestational age range for ACS and tocolytics administration varies across settings and cadre of providers, from as early as 21 weeks to as late as 37 weeks.	[2,6–8,11–18]	12 studies (4 high, 3 moderate, 4 low, 1 very low-quality studies)
**4**	**Providers' perceived risks and benefits**		
**4.1**	**Uncertainties in prescribing and administering ACS for specific populations of women**<br>Quantitative evidence supported the qualitative findings that health providers across settings reported variation on ACS administration practices and beliefs in certain clinical populations. Surveyed providers in quantitative studies had mixed beliefs about the benefits of administration, and desired more research evidence about safety and effectiveness.	[4,13,19–22]	From 6 studies (2 high, 2 moderate, and 2 low quality studies)

#	Summary of Quantitative review findings	Contributing quantitative studies	Quality ratings
4.2	**Scepticism of the evidence base for interventions** Quantitative evidence similarly found that while health providers agreed that ACS are beneficial, some scepticism remained due to fear of birth defects, post-administration side effects, and doubts about benefits.	[4,19,20,23,24]	From 5 studies (1 high, 1 moderate, and 3 low quality studies).
4.3	**Beliefs about risks of interventions** Quantitative evidence supported the qualitative findings regarding concerns about risks after administration of ACS, magnesium sulphate, and tocolytics among health providers.	[4,7,13,19]	From 4 studies (1 high, 1 moderate, 2 low quality studies)
4.4	**Beliefs about risks of interventions – interaction with tocolytics** [no relevant quantitative evidence]	[no relevant quantitative evidence]	[no relevant quantitative evidence]
4.5	**Beliefs about benefits of interventions** Quantitative evidence from health providers supported the qualitative findings regarding recognition of benefits of ACS and magnesium sulphate. However, quantitative evidence from women suggested that women may doubt the benefits of ACS, which can be a barrier to use.	[2,7,12,13,19–21,24,25]	From 10 studies (4 high, 1 moderate, 5 low quality studies)
**5**	**Barriers in administration of interventions**		
5.1	**Uncertainties on when to administer interventions** Quantitative evidence extended understanding of the qualitative evidence, as health providers reported using tocolytics to prolong labour to maximise the effect of ACS, and/or refer women to a higher-level facility.	[4,6–9,12,15,24]	From 8 studies (3 high, 2 moderate, and 3 low quality studies)
5.2	**Time constraints and complexity in prescribing and administering** Quantitative evidence supported the qualitative findings that insufficient time, difficulties in administering ACS, tocolytics, and magnesium sulphate, and high workloads were barriers to use.	[10,13,19,24,26]	From 5 studies (2 high, 1 moderate, 2 low quality studies)
5.3	**Stocking medications in maternity ward** Quantitative evidence extended the understanding of the qualitative evidence that health providers and policymakers believed that ACS and magnesium sulphate were not always available due to insufficient funding and budget allocation resulting in sub-optimal procurement and distribution. Furthermore, health providers may be comfortable prescribing dexamethasone for all women presenting with preterm labour (except for those with signs of infection), and betamethasone only to women with diabetes. In some settings, dexamethasone may be the only corticosteroid available in the hospital, or the only corticosteroid stocked in the maternity setting.	[1,3,4,18,19,23,26,27]	From 8 studies (2 high, 3 moderate, and 3 low quality studies)
5.4	**Regulatory policies and beliefs about prescribing and administering authority** Quantitative evidence extended the qualitative finding that health providers did not have clarity on who was responsible for prescribing and administering ACS and expanding prescription authority may facilitate use. In India, decisions about administering ACS was mostly the role of doctors, but sometimes nurses or auxiliary nurse midwives.	[1,3,28]	From 3 studies (2 moderate and 1 low quality studies)
**6**	**Appropriate settings for administration**		

#	Summary of Quantitative review findings	Contributing quantitative studies	Quality ratings
**6.1**	**Appropriate settings for administration**<br>Quantitative evidence supported the qualitative finding that ACS and tocolytics were mostly used in higher-level health facilities, and that delayed referral is a key barrier. There was also variability regarding the availability of labour and newborn care facilities.	[1,3,7,8]	From 4 studies (1 high, 2 moderate, and 1 low quality studies)
**7**	**Strategies to improve appropriate use**		
**7.1**	**Implementing reminder systems and educational materials**<br>Quantitative evidence supported the qualitative finding that dissemination of educational materials about magnesium sulphate, ACS and tocolytics are useful to health providers and can facilitate appropriate use.	[3,10,26,29–31]	From 6 studies (1 high, 4 moderate, 1 low quality studies)
**7.2**	**Developing reporting indicators and audit and feedback cycles**<br>Quantitative evidence supported the qualitative finding that quality monitoring and improvement systems on ACS are varied across settings. Audit and feedback processes can help to encourage appropriate use of ACS.	[1,3,30]	From 3 moderate studies
**7.3**	**Implementing education and training for health providers**<br>Quantitative evidence supported qualitative evidence that education sessions, workshops, and training sessions for health providers are valuable to encourage use of magnesium sulphate and ACS.	[3,29,30]	From 3 moderate studies
**7.4**	**Appointing “change champions”**<br>Quantitative findings extended qualitative findings that involvement of community-level “change champions”, such as community leaders, can facilitate ACS implementation.	[3,30]	From 2 moderate studies.
**7.5**	**Multi-disciplinary teamwork to improve quality of care**<br>[no relevant quantitative evidence]	[no relevant quantitative evidence]	[no relevant quantitative evidence]
**8**	**Women’s perspectives and experiences**		
**8.1**	**Women and partners’ knowledge of interventions**<br>Quantitative evidence supported the qualitative finding that women’s knowledge about ACS and magnesium sulphate could act as a facilitator or barrier to use, and that misinformation about correct use and poor understanding about benefits can be important barriers.	[4,19,26]	From 3 studies (2 high and 1 moderate quality studies).
**8.2**	**Women learning about preterm birth management**<br>Quantitative evidence supported the qualitative findings that women typically learn about ACS from their health providers, and that some women may not accept ACS and magnesium sulphate due to fears about injections or disapproval from their husband or partner.	[19,22]	From 2 studies (1 high and 1 moderate quality studies).
**8.3**	**Women’s experiences of and concerns about side effects**<br>[no relevant quantitative evidence]	[no relevant quantitative evidence]	[no relevant quantitative evidence]
**8.4**	**Women’s concerns about on impact of interventions on baby**<br>[no relevant quantitative evidence]	[no relevant quantitative evidence]	[no relevant quantitative evidence]

#	Summary of Quantitative review findings	Contributing quantitative studies	Quality ratings
**8.5**	**Regaining control and empowerment**<br>[no relevant quantitative evidence]	[no relevant quantitative evidence]	[no relevant quantitative evidence]
**8.6**	**Trust and relationships between women and health providers**<br>[no relevant quantitative evidence]	[no relevant quantitative evidence]	[no relevant quantitative evidence]
**8.7**	**Seeking support from families and peers**<br>[no relevant quantitative evidence]	[no relevant quantitative evidence]	[no relevant quantitative evidence]
**8.8**	**Coping strategies – reframing experiences**<br>[no relevant quantitative evidence]	[no relevant quantitative evidence]	[no relevant quantitative evidence]
**9**	**Preterm premature rupture of membranes (PPROM) management with antibiotics**		
**9**	**Preterm premature rupture of membranes (PPROM) management with antibiotics**<br>There were no qualitative studies contributing evidence on use of antibiotics for PPROM; however, quantitative studies found that prescribing antibiotics for women with PPROM was common. While some providers reported using antibiotics for PPROM due to evidence of benefit, national guidance, and as Group B Streptococcal Disease (GBS) prophylaxis, some providers reported non-use due to the perception of inconclusive evidence. Antibiotic regimens were highly variable across settings (see S6.1 Appendix).	[8,15,25,32,33]	From 5 studies (1 high, 1 moderate, 2 low and 1 very low-quality studies).

Appendix S6.1 – Different regimens of tocolytics and antibiotics used

Tocolytics regimen
Tocolytic therapy was routinely used by 61% of respondents for preventing preterm birth in women symptomatic for preterm birth. The most common tocolytic agent used was the oxytocin receptor antagonist, atosiban (35%). Two more agents, calcium channel blockers and betamimetics, were used by some respondents (14 and 19%, respectively) [8].<br><br>[Tocolytic] The β-adrenergic drugs were the preferred first choice drug for the suppression of preterm labour (73%). Nifedipine was preferred by 21% of respondents and other drugs were all less than 5%. Again, the β-adrenergic drugs were most used followed by nifedipine [7].<br><br>[Tocolytic] "The data was also analysed based on ‘years since awarded the qualification’. Of those qualified for ≥5 years, 57% would suppress further episodes of preterm labour compared to 44% of those qualified <5 years (P = 0.008). Although β- adrenergic drugs were more commonly used as the first-choice drug by both groups, there was higher use in the ≥5 years qualified group (76%) than the <5 years (61%) group, with nifedipine the opposite (18% in ≥5 years; 34% <5 years). Differences were even more marked in the drug choice for maintenance therapy – β-adrenergic drugs 35% of <5 years and 63% of ≥5 years; nifedipine 54% of <5 years and 20% of ≥5 years [7].<br><br>[Tocolytic] In relation to ‘deliveries per year at your main hospital’, there were several differences in practice. Suppression of preterm labour to prolong pregnancy for the effective administration of steroids was 66% in the smallest units (<500 deliveries) compared to 92% in the larger units (≥500 deliv- eries). A β-adrenergic was the first-choice drug most com- monly used across all units with the highest use in units with <1000 deliveries at 83% decreasing to 57% in units ≥3000 deliveries. The second most commonly used was nifedipine with the opposite trend – that is, used by 39% of respondents in hospitals with ≥3000 deliveries decreasing to 11% in <1000. Trends for the tocolytic drug used in maintenance therapy was similar to the drug of choice for the initial suppression of preterm labour [7].<br><br>[Tocolytic] "The data was also analysed based on ‘years since awarded the qualification’. Of those qualified for ≥5 years, 57% would suppress further episodes of preterm labour compared to 44% of those qualified <5 years (P = 0.008). Although β- adrenergic drugs were more commonly used as the first choice drug by both groups, there was higher use in the ≥5 years qualified group (76%) than the <5 years (61%) group, with nifedipine the opposite (18% in ≥5 years; 34% <5 years). Differences were even more marked in the drug choice for maintenance therapy – β-adrenergic drugs 35% of <5 years and 63% of ≥5 years; nifedipine 54% of <5 years and 20% of ≥5 years [7].<br><br>Differences between the states of Australia were mainly related to the first choice tocolytic drug. In Queensland, 54% of respondents used β-adrenergic drugs and 39% used nifed- ipine whereas in Western Australia, 90% of respondents preferred β-adrenergic drugs and only 6% preferred nifed-ipine. The average usage for the other states was 73% for β-adrenergic drugs and 23% for nifedpine [7].<br><br>Tocolytic agents Of respondents, 81% would consider using tocolytic agents for TPTL in patients carrying HOM gestations (95% CI, 72.4e89.5). The agents most commonly used in HOMs by our

survey population were calcium channel blockers (94%), nonsteroidal antiinflammatory drugs (5%), and nitroglycerin transdermal patch (24%) [6].<br><br>However, the tocolytic prescribed most frequently in 1997–98 was magnesium sulphate (40.6% of respondents), whereas in 2004, it was indomethacin (47.5% of respondents) [9].
**Antibiotics regimen**
Antibiotics were prescribed by 24% of respondents for women symp- tomatic for preterm birth and by one respondent for women asymptomatic for preterm birth. For women symptomatic for preterm birth, erythromycin was the antibiotic most commonly prescribed (16%) and the majority (18%) pre- scribed antibiotics most commonly for 7 days. For women with preterm, pre-labour rupture of membranes (PPROM), the majority of respondents (83 and 83%) routinely pre- scribed antibiotics for women symptomatic and asymp- tomatic for preterm birth, respectively. Erythromycin was the antibiotic most commonly prescribed (50 and 55% in each group, respectively). For the ‘other’ response option, three respondents prescribed metronidazole most com- monly for treatment of women with PPROM [8].<br><br>Antibiotics are used routinely by Australian obstetricians in women with pregnancies complicated with PPROM by 63% (95% CI: 58.8–67.3%). The most commonly used antibiotics are the penicillins ampicillin and amoxicillin (Fig. 1) [15].<br><br>[Antibiotics] A majority of respondents (55%) prescribed multiple agents for prolonged courses. The most common agents included ampicillin (84% or 113 of 134) and erythromycin (29% or 39 of 134); 7 % (10 of 134) used amoxicillin clavulanate [32].

References

[1] Kankaria A, Duggal M, Chauhan A, Sarkar D, Dalpath S, Kumar A, et al. Readiness to Provide Antenatal Corticosteroids for Threatened Preterm Birth in Public Health Facilities in Northern India. Glob Health Sci Pract 2021;9:575–89. https://doi.org/10.9745/GHSP-D-20-00716.

[2] Chollat C, Le Doussal L, de la Villéon G, Provost D, Marret S. Antenatal magnesium sulphate administration for fetal neuroprotection: a French national survey. BMC Pregnancy Childbirth 2017;17:304. https://doi.org/10.1186/s12884-017-1489-z.

[3] Liu G, Segrè J, Gülmezoglu AM, Mathai M, Smith JM, Hermida J, et al. Antenatal corticosteroids for management of preterm birth: a multi-country analysis of health system bottlenecks and potential solutions. BMC Pregnancy Childbirth 2015;15:S3. https://doi.org/10.1186/1471-2393-15-S2-S3.

[4] Aleman A, Cafferata ML, Gibbons L, Althabe F, Ortiz J, Sandoval X, et al. Use of antenatal corticosteroids for preterm birth in Latin America: providers knowledge, attitudes and practices. Reprod Health 2013;10:4. https://doi.org/10.1186/1742-4755-10-4.

[5] Hong JAX, Mathur M. Resident Quality Improvement Project: Antenatal Magnesium Sulfate Protocol for Fetal Neuroprotection in Preterm Births. Obstet Gynecol Int J 2017;Volume 7. https://doi.org/10.15406/ogij.2017.07.00265.

[6] Baker E, Hunter T, Okun N, Farine D. Current practices in the prediction and prevention of preterm birth in patients with higher-order multiple gestations. Am J Obstet Gynecol 2015;212:671.e1-671.e7. https://doi.org/10.1016/j.ajog.2014.12.031.

[7] Cook C-M, Peek MJ. Survey of the management of preterm labour in Australia and New Zealand in 2002. Aust N Z J Obstet Gynaecol 2004;44:35–8. https://doi.org/10.1111/j.1479-828X.2004.00173.x.

[8] Smith V, Devane D, Higgins S. Practices for predicting and preventing preterm birth in Ireland: a national survey. Ir J Med Sci 2011;180:63–7. https://doi.org/10.1007/s11845-010-0604-1.

[9] Hui D, Liu G, Kavuma E, Hewson SA, McKay D, Hannah ME. Preterm labour and birth: a survey of clinical practice regarding use of tocolytics, antenatal corticosteroids, and progesterone. J Obstet Gynaecol Can 2007;29:117–24. https://doi.org/10.1016/S1701-2163(16)32384-2.

[10] Rousseau A, Azria E, Baumann S, Deneux-Tharaux C, Senat MV. Do obstetricians apply the national guidelines? A vignette-based study assessing practices for the prevention of preterm birth. BJOG 2020;127:467–76. https://doi.org/10.1111/1471-0528.16039.

[11] Edmonds BT, McKenzie F, Hendrix KS, Perkins SM, Zimet GD. The influence of resuscitation preferences on obstetrical management of periviable deliveries. J Perinatol 2015;35:161–6. https://doi.org/10.1038/jp.2014.175.

[12] Hueston WJ. Variations between family physicians and obstetricians in the evaluation and treatment of preterm labor. J Fam Pract 1997;45:336–40.

[13] Erickson K, Schmidt L, Santesso DL, Schulkin J, Gregory K, Hobel C. Obstetrician-gynecologists' knowledge and training about antenatal corticosteroids. Obstet Gynecol 2001;97:140–6. https://doi.org/10.1016/s0029-7844(00)01122-4.

[14] Tucker Edmonds B, McKenzie F, Farrow V, Raglan G, Schulkin J. A national survey of obstetricians' attitudes toward and practice of periviable intervention. J Perinatol 2015;35:338–43. https://doi.org/10.1038/jp.2014.201.

[15] Buchanan S, Crowther C, Morris J. Preterm prelabour rupture of the membranes: a survey of current practice. Aust N Z J Obstet Gynaecol 2004;44:400–3. https://doi.org/10.1111/j.1479-828X.2004.00256.x.

[16] Chan KL, Kean LH, Marlow N. Staff views on the management of the extremely preterm infant. Eur J Obstet Gynecol Reprod Biol 2006;128:142–7. https://doi.org/10.1016/j.ejogrb.2006.01.012.

[17] Danerek M, Maršál K, Cuttini M, Lingman G, Nilstun T, Dykes A-K. Attitudes of Swedish midwives towards management of extremely preterm labour and birth. Midwifery 2012;28:e857-864. https://doi.org/10.1016/j.midw.2011.10.009.

[18] Saengwaree P. Changing Physician's Practice on Antenatal Corticosteroids in Preterm Birth. J Med Assoc Thai 2005;88:307–13.
[19] Bousleiman SZ, Rice MM, Moss J, Todd A, Rincon M, Mallett G, et al. Use and attitudes of obstetricians toward 3 high-risk interventions in MFMU Network hospitals. Am J Obstet Gynecol 2015;213:398.e1-11. https://doi.org/10.1016/j.ajog.2015.05.005.
[20] Battarbee AN, Aliaga S, Boggess KA. Management of diabetic women with threatened preterm birth: a survey of Maternal-Fetal Medicine providers. J Matern Fetal Neonatal Med 2020;33:2941–9. https://doi.org/10.1080/14767058.2019.1566307.
[21] Battarbee AN, Clapp MA, Boggess KA, Kaimal A, Snead C, Schulkin J, et al. Practice Variation in Antenatal Steroid Administration for Anticipated Late Preterm Birth: A Physician Survey. Am J Perinatol 2019;36:200–4. https://doi.org/10.1055/s-0038-1667028.
[22] Aghajafari F, Murphy K, Ohlsson A, Amankwah K, Matthews S, Hannah ME. Multiple versus single courses of antenatal corticosteroids for preterm birth: a pilot study. J Obstet Gynaecol Can 2002;24:321–9. https://doi.org/10.1016/s1701-2163(16)30625-9.
[23] Capeless EL, Mead PB. Management of preterm premature rupture of membranes: lack of a national consensus. Am J Obstet Gynecol 1987;157:11–2. https://doi.org/10.1016/s0002-9378(87)80335-6.
[24] Vargas-Origel A, León Ramírez D, Zamora-Orozco J, Vargas-Nieto MA. [Prenatal corticosteroids. Use and attitudes of the gynecology-obstetrics medical staff]. Ginecol Obstet Mex 2000;68:291–5.
[25] Wilson B, Thornton JG, Hewison J, Lilford RJ, Watt I, Braunholtz D, et al. The Leeds University Maternity Audit Project. Int J Qual Health Care 2002;14:175–81. https://doi.org/10.1093/oxfordjournals.intqhc.a002609.
[26] Gatman K, May R, Crowther C. Survey on use of antenatal magnesium sulphate for fetal neuroprotection prior to preterm birth in Australia and New Zealand - Ongoing barriers and enablers. Aust N Z J Obstet Gynaecol 2020;60:44–8. https://doi.org/10.1111/ajo.12981.
[27] Smith JM, Gupta S, Williams E, Brickson K, Ly Sotha K, Tep N, et al. Providing antenatal corticosteroids for preterm birth: a quality improvement initiative in Cambodia and the Philippines. Int J Qual Health Care 2016;28:682–8. https://doi.org/10.1093/intqhc/mzw095.
[28] Tucker Edmonds B, McKenzie F, Panoch JE, Barnato AE, Frankel RM. Comparing obstetricians' and neonatologists' approaches to periviable counseling. J Perinatol 2015;35:344–8. https://doi.org/10.1038/jp.2014.213.
[29] Bain E, Bubner T, Ashwood P, Crowther CA, Middleton P, WISH Project Team. Implementation of a clinical practice guideline for antenatal magnesium sulphate for neuroprotection in Australia and New Zealand. Aust N Z J Obstet Gynaecol 2013;53:86–9. https://doi.org/10.1111/ajo.12008.
[30] McGoldrick EL, Brown JA, Groom KM, Crowther CA. Investigating antenatal corticosteroid clinical guideline practice at an organisational level. Aust N Z J Obstet Gynaecol 2017;57:25–32. https://doi.org/10.1111/ajo.12564.
[31] Burhouse A, Lea C, Ray S, Bailey H, Davies R, Harding H, et al. Preventing cerebral palsy in preterm labour: a multiorganisational quality improvement approach to the adoption and spread of magnesium sulphate for neuroprotection. BMJ Open Qual 2017;6:e000189. https://doi.org/10.1136/bmjoq-2017-000189.
[32] Glass NE, Schulkin J, Chamany S, Riley LE, Schuchat A, Schrag S. Opportunities to reduce overuse of antibiotics for perinatal group B streptococcal disease prevention and management of preterm premature rupture of membranes. Infect Dis Obstet Gynecol 2005;13:5–10. https://doi.org/10.1080/10647440400028144.
[33] Kenyon S, Pike K, Jones D, Brocklehurst P, Marlow N, Salt A, et al. Has publication of the results of the ORACLE Children Study changed practice in the UK? BJOG 2010;117:1344–9. https://doi.org/10.1111/j.1471-0528.2010.02661.x.
