# Supplementary material for: Factors influencing appropriate use of interventions for management of women experiencing preterm birth: A mixed-methods systematic review and narrative synthesis
Source: PLoS Med. 2022 Aug 23;19(8):e1004074. doi: 10.1371/journal.pmed.1004074 (PMC9398034; doi:10.1371/journal.pmed.1004074)
Supplement: S7 Appendix — (PDF) [file pmed.1004074.s007.pdf]

Appendix S7. Summary of Study Designs and Type of Interventions

Methodology	Total studies	ACS	Tocolytics	MgSO4 for fetal neuroprotection	Antibiotics for PPROM
**Quantitative**	32	24	7	5	5
**Qualitative**	11	7	6	2	0
**Mixed methods**	3	1	0	2	0
**Total**	46	32	13	9	5
