# Supplementary material for: Factors influencing appropriate use of interventions for management of women experiencing preterm birth: A mixed-methods systematic review and narrative synthesis
Source: PLoS Med. 2022 Aug 23;19(8):e1004074. doi: 10.1371/journal.pmed.1004074 (PMC9398034; doi:10.1371/journal.pmed.1004074)
Supplement: S8 Appendix — (PDF) [file pmed.1004074.s008.pdf]

S8 Appendix. Development of Themes

Final themes	Emergent themes	Coverage of topic scope
**Inaccurate assessment of gestational age**	Inaccurate assessment of gestational age	Ensuring the right women receive the right interventions
**Inconsistent practice guidelines**	Inconsistent practice guidelines	Ensuring the right women receive the right interventions
**Variable knowledge about the interventions**	Health providers' knowledge of the interventions	Ensuring the right women receive the right interventions
	Knowledge about optimal gestational age for intervention administration	Ensuring the right women receive the right interventions
**Providers' perceived risks and benefits**	Uncertainties in prescribing and administering ACS for specific populations of women	Ensuring the right women receive the right interventions
	Scepticism of the evidence for interventions	Ensuring the right women receive the right interventions
	Beliefs about risks of interventions	Ensuring the right women receive the right interventions
	Beliefs about risks of interventions – interaction with tocolytics	Ensuring the right women receive the right interventions
	Beliefs about benefits of interventions	Ensuring the right women receive the right interventions
**Barriers in administration of interventions**	Uncertainties on when to administer interventions	Appropriate duration of exposure
	Time constraints and complexity in prescribing and administering	Appropriate duration of exposure
	Stocking medications in maternity ward	Appropriate duration of exposure
	Regulatory policies and beliefs about prescribing and administering authority	Appropriate settings for administration
**Appropriate settings for administration**	Appropriate settings for ACS administration	Appropriate settings for administration
**Strategies to improve appropriate use**	Implementing reminder systems and educational materials	Ensuring the right women receive the right interventions
	Developing reporting indicators and audit and feedback cycles	Ensuring the right women receive the right interventions
	Implementing education and training for health providers	Ensuring the right women receive the right interventions
	Appointing "change champions"	Ensuring the right women receive the right interventions
	Multi-disciplinary teamwork to improve quality of care	Ensuring the right women receive the right interventions
**Women's perspectives and experiences**	Women and partners' knowledge of interventions	Ensuring the right women receive the right interventions
	Women learning about preterm birth management	Ensuring the right women receive the right interventions
	Women's experiences of and concerns about side effects	Ensuring the right women receive the right interventions

Final themes	Emergent themes	Coverage of topic scope
	Women’s concerns about on impact of interventions on baby	Ensuring the right women receive the right interventions
	Regaining control and empowerment	N/A
	Trust and relationships between women and health providers	Ensuring the right women receive the right interventions
	Seeking support from families and peers	Appropriate duration of exposure
	Coping strategies – reframing experiences	Appropriate duration of exposure
