# Supplementary material for: Factors influencing appropriate use of interventions for management of women experiencing preterm birth: A mixed-methods systematic review and narrative synthesis
Source: PLoS Med. 2022 Aug 23;19(8):e1004074. doi: 10.1371/journal.pmed.1004074 (PMC9398034; doi:10.1371/journal.pmed.1004074)
Supplement: Attachment — Submitted filename: ACS+ review_Response to reviewers_05-23-2022.docx [file pmed.1004074.s009.docx]

Dear Editors and Reviewers,

Many thanks for the constructive feedback on our paper. We have addressed the point-by-point feedback below and with revisions in a tracked changes version of the manuscript. We appreciate this feedback and believe that it has strengthened our paper.

We also note that we have conducted an update of the 4 database searches given that the previous searches were run in November 2021. All searches are now up-to-date to May 16, 2022.

Sincerely,

Meghan Bohren, on behalf of the authors

| Reviewer feedback | Author’s response |
| --- | --- |
| EDITORAL COMMENTS |  |
| I disagree with reviewer 2's perspective re Ultrasound assessment of gestational age. I do not see this recommendation as problematic in this context and thinks the authors discuss this appropriately. | Thank you for your feedback, we have not made any revisions based on this comment from reviewer 2. |
| What geographies/studies might be associated with the different barrier types? For example, it would be better to say that the limited diagnostics for gestational age means that rural areas have inability to determine gestational age, rather than to say that gestational age assessment is a barrier. Maybe there could be a comment on what might be missed and why/how. This is a window not of exactly where the problems are but where the literature has located the problems. Often, for example, structural things are harder to study and get swept under the carpet. | Thank you for this comment. We agree with you that there may be different barriers associated with aspects related to the study context (e.g. different geographies). In the qualitative findings, information on the study context and study populations is considered as part of the “Relevance” assessment within GRADE-CERQual, and is reported comprehensively in S5 Evidence Profile for each review finding – and in summary form in Table 3 – Summary of qualitative findings. However we note that we are limited in how far we can extrapolate to describe e.g. specific geographies that may be most or more impacted, based on the data from the included studies, much of which is thin data related to study context. We have addressed this comment in a re-write of the Discussion section, when taken together with other editorial and reviewer feedback. Please see revisions in the discussion section. |
| General comments:  Throughout the paper, please adapt reference call-outs to the following style: "... 24 to 35 weeks gestational age [12,18,19]." (noting the absence of spaces within the square brackets). | We have now changed the parentheses into square brackets as requested. |
| Please remove formatting such as emboldening and italicisation in the main text of your manuscript (particularly in the Results sections) | We have removed all formatting from the text of the manuscript, and the only remaining formatting is related to section headers. |
| Abstract:  Please report your abstract according to PRISMA for abstracts, following the PLOS Medicine abstract structure (Background, Methods and Findings, Conclusions) - <http://www.plosmedicine.org/article/info:doi/10.1371/journal.pmed.1001419>  Abstract Methods and Findings:  Please provide the number of studies included/number of participants, types of study designs included (e.g. RCTs, cohort studies, etc), eligibility criteria, and synthesis/appraisal methods (including evaluation of study quality and risk of bias).  In the last sentence of the Abstract Methods and Findings section, please describe 2-3 of the main limitations of the study's methodology.  Abstract Conclusions:  Please begin your Abstract Conclusions with "In this study, we observed ..." or similar, to summarize the main findings from your study, without overstating your conclusions. Please emphasize what is new and address the implications of your study, being careful to avoid assertions of primacy. | Thank you – we have updated the abstract based on the PRISMA for abstracts structure and editorial feedback. Please see the revised abstract in the manuscript file (page 2). |
| Author Summary:  At this stage, we ask that you include a short, non-technical Author Summary of your research to make findings accessible to a wide audience that includes both scientists and non-scientists. The Author Summary should immediately follow the Abstract in your revised manuscript. This text is subject to editorial change and should be distinct from the scientific abstract. Please see our author guidelines for more information: <https://journals.plos.org/plosmedicine/s/revising-your-manuscript#loc-author-summary>  In the final bullet point of ‘What Do These Findings Mean?’, please describe the main limitations of the study in non-technical language. | Thank you – please see the new section “Author summary” on page 3 of the manuscript file. |
| Introduction:  If there has been a systematic review of the evidence related to your study (or you have conducted one), please refer to and reference that review. | There was previously no systematic review of the evidence related to our study; in our study, we conducted a systematic review. No revisions made. |
| Methods:   1. Please state early in the Methods section whether any reported analyses differed from those that were planned in your PROSPERO protocol. Changes in the analysis, including those made in response to peer review comments, should be identified as such in the Methods section of the paper, with rationale. If a reported analysis was performed based on an interesting but unanticipated pattern in the data, please be clear that the analysis was data-driven. 2. Thank you for providing a completed PRISMA checklist. Please add the following statement to the Methods: "This study is reported as per the Preferred Reporting Items for Systematic Reviews and Meta-Analyses (PRISMA) guideline (S1 Checklist).” 3. Please consider including PubMed in your search. 4. Please update your search to the present time. We require that SRs are updated to within roughly 6 months of the expected publication date. | 1. There were no reported analyses that differed from those planned in the PROSPERO protocol. No revisions made. 2. Thank you – we have added this statement verbatim in the first section of the methods. 3. Thank you for your feedback. We note that PubMed is an interface used to search Medline, and all articles indexed in Medline are also indexed in PubMed. We have searched MEDLINE using the Ovid interface, which allows for a more focused search compared to using the PubMed interface. Had we searched using PubMed using the same search strategy, we would have had the same records returned. 4. Thank you – we have updated the searches to 16 May 2022 and no additional relevant references were included. |
| Results:  Please include a table showing the characteristics of the included studies, including data from the original studies on the participants, interventions and outcomes, which should be incorporated into the main paper (this is currently Appendix S7). | We have moved the Characteristics of included studies from Appendix S7 to Table 2 in the manuscript. |
| Discussion:   1. Please present and organize the Discussion as follows: a short, clear summary of the article's findings; what the study adds to existing research and where and why the results may differ from previous research; strengths and limitations of the study; implications and next steps for research, clinical practice, and/or public policy; one-paragraph conclusion. 2. Please remove all subheadings within your Discussion e.g. Summary of main results 3. Please move the section on ‘Implications for practice’ in its current form to the supplementary information, and discuss the implications of your findings in prose form within the main text of the Discussion Line 760 - please temper assertions of primacy by adding ‘to the best of our knowledge’ or similar | Please see revisions throughout the Discussion section, which are too long to paste here. We have moved the ‘Implications for practice’ questions to Appendix S9. |
| Figures:   1. Please define all abbreviations used in the figure legend of each figure. 2. Please consider avoiding the use of red and green in order to make your figure more accessible to those with colour blindness. | 1. Thank you, we have defined all abbreviations in the figure legend, which is in the manuscript file just under the title of each figure. 2. We have revised all the figures by using color blind friendly palette generated by https://coolors.co/.. |
| Tables:  Please define all abbreviations used in the table legend of each table. | Please see revisions in Table 1 and Table 3 footnotes. |
| References:  Please ensure that journal name abbreviations match those found in the National Center for Biotechnology Information (NCBI) databases (<http://www.ncbi.nlm.nih.gov/nlmcatalog/journals>), and are appropriately and consistently formatted and capitalised e.g. refs 3, 5, 7, etc.  Please also see <https://journals.plos.org/plosmedicine/s/submission-guidelines#loc-references> for further details on reference formatting.  Where website addresses are cited, please specify the date of access. | We have revised journal abbreviations based on NCBI databases. Our references included journal articles, websites, and reports/guidelines and we have checked that formatting following recommendations from PLOS website, including having URL and date access for websites. Tracked changes were not recorded when editing references as reference manager was used. |
| Supplementary files:  Please see <https://journals.plos.org/plosmedicine/s/supporting-information> for our supporting information guidelines. | We have checked the guidelines and have prepared our supporting information accordingly. |
| REVIEWER 1 |  |
| Reviewer #1: This is a systematic review of both quantitative and qualitative studies to identify factors influencing appropriate use of interventions for preterm birth. The study, ambitiously has five objectives, to 1) explore perceptions, preferences and experiences of women, partners, health providers and other relevant stakeholders on the use of four preterm birth interventions; 2) explore how health workers identify women at risk of preterm birth, including assessment of gestational age, identifying signs of maternal infection, and recognizing risk of preterm birth; 3) identify factors affecting administration and duration of exposure of the four interventions; 4) explore whether the factors affecting appropriate use differ across types of health facilities; and 5) use Theoretical Domains Framework (TDF) and Capability, Opportunity, and Motivation (COM-B) model of behavior change to explore potential strategies in improving appropriate use and scale up of the four interventions. Overall, I think this is an ambitious and comprehensive study trying to provide useful evidence on barriers and facilitators of interventions of preterm birth and give suggestion on implementation strategies. Here are my specific comments: | Thank you. |
| 1. Line 116-117: Why did you only include studies with cross-sectional and mixed-methods approaches for data collection? Why not consider studies collecting data using other methods, such as surveillance, registration, or hospital/facility record? Also, Why not included studies of interventions that increase the use of those preventative measures. Those interventions may target specific factors that prohibit of promote use of the four interventions. | Thank you for this comment. The topic of interest in our review was factors (barriers or enablers) affecting use of ACS, tocolytics, magnesium sulphate, and antibiotics, and the type of included studies were specified as the most appropriate designs to answer these questions. Our search strategy had no limitations on methodological study designs, and we therefore screened studies such as surveillance and hospital/record – none of these studies were ultimately included because they did not report on factors affecting use – typically these studies focused on coverage. Studies that aimed to increase use of the 4 interventions were eligible for inclusion if they also reported on factors affecting use – typically this might be found in something like formative research, process evaluation or impact evaluation. These types of studies were included – such as Smith 2016 and Teela 2015. No revisions made. |
| 2. Line 127-129: Why did you only included studies exploring perspectives of people? Why not studies exploring factors measured more objectively such as level of facility, quantity, and quality of health personnel and other resources? | Thank you for this comment. The factors listed by the reviewer (e.g. level of facility, quantity, and quality of health personnel and other resources) were assessed as part of objectives 3 and 4 of our review. Studies that met the inclusion criteria and contribute evidence regarding these domains were included and are reflected in our findings. For example, finding 5.3 regarding stocking medications in the maternity ward, 6.1 about appropriate settings for ACS administration, and findings related to healthcare provider knowledge and limitations in findings 3.1, 3.2, 4.1, 4.2, 4.3, 4.4 and 4.5. No revisions made. |
| Line 133: Why did you not use PubMed? | We note that PubMed is an interface used to search Medline, and all articles indexed in Medline are also indexed in PubMed. We have searched MEDLINE using the Ovid interface, which allows for a more focused search compared to using the PubMed interface. Had we searched using PubMed using the same search strategy, we would have had the same records returned. |
| Line 137: how did you decide to only look at the first 10 pages? | Most search engine users would look the first 3-5 pages search results as the higher the number of pages we go, the more irrelevant the results would be. However, we decided to go up to page 10 to capture a more comprehensive results on each intervention. Previous studies using google search also looked at results up to 10 pages for similar reasons (Xiang et al 2010), another study has even just used 20 first links regardless number of pages (Yamaguchi et al 2020). |
| Line 173: How did you select the initial 6 studies for developing the codebook? What are the specific criteria? | We selected the 6 studies based on the thickness of data, which is a term in qualitative research to refer to the richness of the data, or how thick of a description in the included studies. Studies with “thick” data typically have a level of complexity that tells more involved stories, with longer quotes from participants and author interpretation of the data, that allows a reader to better understand the how and the why. We selected the 6 studies based on the thickness of data, which we have mentioned in line 209-210 (tracked changes version). No revision made. |
| Line 176-178: The methods of synthesizing qualitative data need to be reviewed by an expert of qualitative analysis. | Thank you, we trust that this has been accounted for in the editorial process. |
| Line 179-181: Why not the other why around? Why qualitative data were not used to extend quantitative evidence? To me, qualitative data should be much broader than quantitative data, especially given that you have more quantitative studies (32) than qualitative studies (11). | Thank you for this comment. Given the heterogeneity in the quantitative data (very different outcome measures, study designs), we were not able to conduct a quantitative meta-analysis – which we expected and specified in our review protocol. Therefore, the most appropriate approach for analyzing the quantitative data is a narrative approach, which we have used. We note that developing categories by which to describe the quantitative data in a narrative review is an important step, and in the case of this review, we used the qualitative data to develop these themes or categories. Starting with the qualitative data also gave us a richer picture of the available evidence in more detail (e.g. multiple pages of author interpretations and participant quotations) compared to quantitative data (e.g. typically 1-2 measures with limited contextualization). We developed this analysis approach based on Popay 2006 guidance on narrative synthesis in systematic reviews (ref 31). No revisions made. |
| Line 179-187: I think you may need some more explanation on how you mapped the quantitative data and qualitative findings and on how you mapped the findings to the TDF and COM-B. | Please see revisions in text to clarify and provide an example (line 229-232):  *We firstly mapped facilitators and barriers to the fourteen domains of the TDF, then mapped to the three COM-B domains (ref). For example, healthcare provider ‘lack of awareness on ultrasound dating for gestational age’ mapped to the Capability-Knowledge domain. After mapping we identified potential strategies from each of three COM-B domains to promote the two intended behaviours (ref).* |
| Line 188: provides. | Thank you – we have revised (line 225-226). |
| Line 208-218: may put some of the details to the appendix here. | Based on editorial feedback, we have moved Table 2 – Characteristics of included studies to the main manuscript. We have left the descriptions of the included studies (now lines 249-279) in the main manuscript text per PRISMA reporting guidance. No revision made. |
| Lastly, I recommend the study be reviewed by experts on qualitative analysis and on preterm births. | Thank you, we trust that this has been accounted for in the editorial process. |
| REVIEWER 2 |  |
| The authors have done an interesting review, that is much needed. The authors provide impressive and interesting tables and figures. The manuscript is dense, and as I understand it is based on work done for a WHO recommendation. However, if this is to be an article, rather than a report, I find the text in the results section difficult to go through. There is quite some repetition and I wonder if this could be better covered by the tables provided, with the results section in the text to be reserved for contextualising these results. | Thank you. We have made some revisions throughout to improve readability, particularly in the discussion section. Please see changes throughout. |
| I have one major concern, which might be resolved by simple changes in language - but might also more complex as this has to do with the near absence of focus on context, which is only given some attention in the discussion. The authors have a clear focus on the importance of GA dating via ultrasound, and in line 643 other methods of determining GA are deemed Inaccurate. Yet, the reality is that in the majority of countries in LMIC setting this is the Only method available for GA dating. And while this might indeed be LESS accurate than ultrasound dating, this does not mean it cannot be useful, in particular for promoting the very much needed interventions this review is discussing. I am aware the WHO in their ANC guidelines recommend early ultrasound assessment for all women, and the current recommendation for interventions for preterm birth likely have to refer to these, However, for this article I find this problematic. And you might perhaps see this as a form of bias. At the bear minimum the recommendation and therefore this article needs to offer LMIC that do not have ability to provide all women with an early scan an alternative. Table 1 stating that WHEN ACS can be provided is only when GA is accurately assessed through ultrasound, makes the rest of the text and review completely inaccessible and useless for countries that won't be able to live up to this recommendation. And this means many women and newborns that could potentially benefit from the interventions in this review, will miss these opportunities. | Thank you for your feedback. Per the editor’s comment, we have not made any revisions regarding the presentation of ultrasound for GA dating. We note that trials of ACS efficacy were predominantly from high income countries with ultrasound availability. However, the Antenatal Corticosteroid Trial (ACT) (Althabe 2015) showed potential harm when less accurate methods of GA estimation (including uterine fundal height assessment) were applied, and several ineligible women (and their babies) were inadvertently exposed to ACS. In the light of trial evidence of ACS efficacy from high-resource settings where accurate GA determination were possible, and unexpected results of harm from a population-based study where only a fraction of women had GA determined by ultrasound (Althabe 2015), the WHO 2015 overarching ACS recommendation stipulates ultrasound GA confirmation as one of the pre-requisites for ACS use. WHO had made this recommendation acknowledging the limitations of having ultrasound GA consistently for all women in LMICs, but placed higher emphasis on addressing safety concerns associated with ACS use in the context of inaccurate GA determination. A subsequent landmark trial, the WHO ACTION-I trial, conducted in 5 LMICs (Bangladesh, India, Kenya, Nigeria and Pakistan) demonstrated benefits of ACS in the context of ultrasound-based GA determination for all women treated with ACS, contrary to the findings of ACT, where the majority of women had their GA determined using less accurate method. There are now several handheld/portable ultrasound devices which are being promoted for use by several health care cadre (including nurses and midwives) to improve and scale up basic ultrasound services in LMICs. |
| A reflection on the barriers of global guidelines - that are very much based on evidence generated in high income settings and how these are not always transferable to LMIC settings could strengthen the review, in particular as the authors emphasise the importance of implementation science. Stressing the importance of the need for contex-adapting guidelines - and thereby perhaps creating a platform for discussing optimal treatment - is recommended. Perhaps in some countries this means focussing on those women that will have an expected pre-term birth (women with Pre eclampsia for example). This also means guidelines need to be integrated or connected in some way. There are major research gaps in this field in LMIC setting and the authors could stress the importance of conducting studies in these settings. | Thank you, we strongly agree with your comment. Please see revised Discussion section (too lengthy to paste here) to better reflect the dynamics of translating global guidelines into practice, critical gaps around implementation research, and particular research and practice gaps in LMIC settings. |
| REVIEWER 3 |  |
| Thank you for the opportunity to review an important and high-quality piece of work, thus I have only minor comments/suggestions for publication. Authors conducted a complex mixed methods review and narrative synthesis and assessed contextual factors and implementation strategies influencing use of antenatal corticosteroids, tocolytics, magnesium sulphate, antibiotics, for management of women at risk of preterm birth. | Thank you. |
| 1) This is an important implementation research question in the maternal public/global health and preterm birth field and authors made it very clear in the background section. Perhaps authors could add in the background the rational for selecting those 4 (just clinical) interventions that are used during pregnancy or just before preterm birth (2015 WHO guideline on recommendations on interventions to improve preterm birth outcomes is only mentioned in methods). Also, a more recent Cochrane review also found other clinical interventions (e.g. screening for lower genital tract infections; zinc supplementation, cervical stitch for women at high risk of preterm birth) had a clear benefit on preterm birth outcomes (Medley et al 2018 - Interventions during pregnancy to prevent preterm birth: an overview of Cochrane systematic reviews). I think the four interventions selected by authors will be targeting a specific population group, pregnant women at risk of preterm birth (who may or may not necessarily experience a preterm birth at the end). | Thank you for your comment, we agree the implementation and scale-up of these four interventions is critical for global maternal and newborn health, and that there are also other interventions besides these four that can make an impact. The selection of the four interventions is, as Reviewer 3 notes, based on the 2015 WHO guideline on recommendations on interventions to improve preterm birth outcomes, which focuses on “tertiary interventions” that are most impactful in terms of improving newborn survival. The WHO ACTION-I trial has shown that ACS alone is the most impactful in terms of improving newborn survival (Number Needed to Treat=25). There are other primary interventions (e.g. lifestyle improvements or pre-pregnancy cautions) that have mixed results and are difficult to implement on a large scale, as well as secondary interventions (e.g. cervical cerclage) which can only be provided to selected women in highly-resourced hospital settings, meaning that they are not interventions that can be readily taken to scale. Reviewer 3 is correct in that the 2015 WHO Guideline and our current review focus on “women who have an imminent risk of preterm labour”, whereas the aforementioned primary and secondary interventions target broader populations of women, most of whom may indeed not experience preterm birth. We have clarified this in the Introduction, lines 117-121:  *While there are other primary interventions (e.g. smoking cessation programmes) and secondary interventions (e.g. cervical cerclage, progestational agents) for preterm birth, the 2015 World Health Organization (WHO) recommendations on interventions to improve preterm birth outcomes specifies that the most beneficial set of maternal interventions are those aiming to improve outcomes for preterm babies when preterm birth is inevitable (e.g. ACS, magnesium sulphate, antibiotics) (ref).* |
| 2) The methods section is very comprehensive, and authors report the review in accordance with recommended guidelines. There are not important deviations from the prospero registration. It is innovative to see how qualitative and quantitative data are mapped together and how identified contextual factors are then mapped to TDF and COM-B models of behaviour change to identify strategies - authors could explain briefly why they selected those two models and not others and how/why those two behaviours (appropriate use by providers, acceptability by women) were chosen for the purposes of the mapping? | Thank you for your comment. We have made revisions to explain the use of the models in lines 225-233 (tracked changes version):  *COM-B is a comprehensive behaviour change model which provides a framework to assess three fundamental conditions that must be understood and addressed to promote behaviour change. We defined two behaviours for the purposes of the mapping, based on the scope of the review questions (Fig 1) and previously known threats to implementation: 1) appropriate use of the four interventions by providers; 2) acceptability of the four interventions by women. We firstly mapped facilitators and barriers to the fourteen domains of the TDF, then mapped to the three COM-B domains (REF). For example, healthcare provider ‘lack of awareness on ultrasound dating for gestational age’ mapped to the Capability-Knowledge domain. After mapping we identified potential strategies from each of three COM-B domains to promote the two intended behaviours (ref).* |
| 3) Findings are very clearly presented in the text ,tables. Since most of the included studies in the review are from HIC, would it be useful for readers to know where the data from the overarching themes and strategies came from and understand variations from different country income levels? (e.g. in the 'Limitations about determining gestational age' - authors do mention data from LMIC but not in other themes?). Just a suggestion but I understand it may not be possible for pragmatic purposes! And this is part of the discussion. | Thank you for this. Information on the study context and study populations is considered as part of the “Relevance” assessment within GRADE-CERQual, and is reported comprehensively in S5 Evidence Profile for each review finding – and in summary form in Table 3 – Summary of qualitative findings. Please see revisions in the Discussion section to better account for these differences across contexts (noting that the revisions are too lengthy to paste here). |
| 4) Figure 5: Mapping factors affecting health providers' appropriate use of interventions for preterm birth management using COM-B - Not sure why implementation strategies are inside the facilitator boxes (2nd column)? Would it be more appropriate to add a third column with implementation strategies (barriers/facilitators/implementation strategies) - you would expect both barriers and facilitators to inform implementation strategies? Eg confusion about different courses, doses etc can be a barrier and can inform eg the development of education and training for care providers (to address that barrier). | Figure 5 depicts the identified factors (barriers, facilitators, or mixed barriers/facilitators) identified in the review. Ideas for how to address these barriers or reinforce facilitators from the perspective of the research team are not described in this figure. We think that this comment is in relation to some of the ‘facilitators’ sounding similar to an ‘implementation strategy’, but note that these ‘facilitators’ are from the perspectives of the participants from the included studies – not our research team. No revision made. |
| 5) Discussion, limitations and implications for practice and research are very well presented. Minor comment on wording: the review sometimes uses 'preterm birth interventions' or uses preterm birth indistinctively with preterm labour and this may need to be revised e.g. a woman may go into preterm labour and have some interventions for management (thus at risk of preterm birth) but may not end up experiencing a preterm birth. I am not sure how appropriate is to use 'threatened preterm birth' (but threatened preterm labour) or 'preterm birth interventions' (but eg. interventions during pregnancy to reduce preterm birth) | Thank you for this helpful feedback. We have revised the whole Discussion section based on editorial feedback. Please see revisions in the text of the Discussion section (as too lengthy to paste here). We have also made minor revisions to the abstract and introduction throughout to clarify that we are referring to “interventions to improve preterm birth outcomes and/or management”. |
| 6) It is clear this study findings will be helpful for policy makers and implementers who are planning policy to implement and scale up clinical interventions to reduce preterm birth (I think important abstract findings only mention barriers maybe due to word count? I think facilitators and strategies are also very important). Congratulations to authors for such a great work! | Thank you, we hope so too! |
