# Supplementary material for: Factors influencing appropriate use of interventions for management of women experiencing preterm birth: A mixed-methods systematic review and narrative synthesis
Source: PLoS Med. 2022 Aug 23;19(8):e1004074. doi: 10.1371/journal.pmed.1004074 (PMC9398034; doi:10.1371/journal.pmed.1004074)
Supplement: Attachment — Submitted filename: PTB interventions_Response to Reviewers_06-29-2022.docx [file pmed.1004074.s010.docx]

| Reviewer feedback | Author’s response |
| --- | --- |
| EDITORAL COMMENTS |  |
| 1. Abstract - please trim down the background section to 3-4 sentences. | Thank you for your feedback. There are only 4 sentences on the background section of abstract. We assumed that editors referred to methods and findings instead of background, which we have revised. |
| 2. Author summary – please trim the 3rd bullet under “What did the researchers do and find?” This should contain at most 4 sentences. | Thank you for your feedback. We have trimmed some words on the third bullet points under this section. |
| 3. Please use a uniform black color for main text, headings, and subheadings, table (lines and text) | Thank you, we have changed all colors to black. |
| 4. PRISMA and ENTREQ checklists- when completing the checklists, please use both section and paragraph numbers, rather than section headers only. | Thank you, we have included section and paragraph numbers on PRISMA and ENTREQ checklists documents. |
| 5. Please remove the ‘Sources of funding,” “Competing interest” statements from the end of the main text. In the event of publication, this information will be published as metadata based on your responses to the submission form. | Thank you, we have removed these two sections on the manuscript. |
| REVIEWER 1 |  |
| Reviewer #1: I think the authors have answered my questions and have addressed concerns of mine and of other reviewers adequately. Once the manuscript has met all the editorial requirements, I think it can be accepted. Well done and congratulations to the authors. | Thank you for your very kind response. |
| REVIEWER 3 |  |
| Reviewer #3: Thank you again for the opportunity to review this revision. I think authors have addressed well most comments and the manuscript has significantly improved. I would suggest some minor changes before accepting for publication.  Abstract and author summary: the paper is assessing assess factors (barriers and facilitators) affecting the appropriate use of 4 key interventions to prevent or manage preterm birth and identifying potential strategies - why findings in these two sections present only barriers? Suggest briefly including findings on all, barriers, facilitators and potential strategies. | Thank you for your feedback. Based on the editorial feedback to shorten the abstract and author summary, we have not added the facilitators and potential strategies and instead focusing on the barriers to emphasize on what should be improved to ensure appropriate use of the four interventions. |
| I am still trying to understand Figure 4 and 6 and I think a small revision will be helpful here - authors report they map factors (barriers, facilitators/mixed evidence) affecting health providers' appropriate use and women's acceptance of interventions for preterm birth management using behavioural frameworks. However, figures can be misleading by reporting 'implementation strategies' within the 'facilitators' boxes. I presume those 'facilitators' and 'implementation strategies' were from the perspectives of the participants from the included studies (it is surprising to see that no included study suggested implementation strategies for specific barriers?). However, in the field of implementation research, there is a clear distinction between 'barriers and facilitators' (aka 'determinants of implementation or any contextual factors external to an intervention that affect implementation success) and 'implementation strategies' (methods/techniques used to enhance the adoption, implementation, and sustainability of an intervention). Thus, barriers & facilitators/ contextual factors inform the appropriate selection of implementation strategies. | Thank you for your helpful feedback. You are right that the facilitators which we mentioned as “implementation strategies” were identified as such from the included studies – not developed by the review authors using an implementation science approach. To avoid confusion, we have deleted the heading “implementation strategies” and merged it with other facilitatorsin Fig 4 and Fig 6. |
| I understand other reviewers suggested to shorten the discussion section and it is now more comprehensive and less repetitive. But not sure about moving the 'implications for practice and research' to an appendix, this is a very important section of any discussion and a short summative paragraph in the main paper will be extremely valuable. Good luck! | Thank you for your kind comment. To ensure readability of the discussion while emphasizing on our implications for practice and research, we have moved this section as a table on the manuscript instead of supplementary file. Please see Table 4. |
